# Clinical Trial of Prophylactic Extended-Field Carbon-Ion Radiotherapy for Locally Advanced Uterine Cervical Cancer (Protocol 0508)

**DOI:** 10.1371/journal.pone.0127587

**Published:** 2015-05-20

**Authors:** Masaru Wakatsuki, Shingo Kato, Hiroki Kiyohara, Tatsuya Ohno, Kumiko Karasawa, Tomoaki Tamaki, Ken Ando, Hirohiko Tsujii, Takashi Nakano, Tadashi Kamada, Makio Shozu, The Working Group of the Gynecological Tumor

**Affiliations:** 1 Research Center for Charged Particle Therapy, National Institute of Radiological Sciences, Chiba, Japan; 2 Department of Radiation Oncology, Saitama Medical University International Medical Center, Saitama, Japan; 3 Department of Radiation Oncology, Gunma University Graduate School of Medicine, Maebashi, Gunma, Japan; 4 Gunma University Heavy Ion Medical Center, Gunma University Graduate School of Medicine, Maebashi, Gunma, Japan; 5 Department of Radiation Oncology, Gunma Prefectural Cancer Center, Ota, Gunma, Japan; 6 Department of Reproductive Medicine, Graduate School of Medicine, Chiba University, Chiba, Japan; Institut Gustave Roussy, FRANCE

## Abstract

To evaluate the efficacy and the toxicity of prophylactic extended-field carbon-ion radiotherapy (C-ion RT, Protocol 0508) for locally advanced squamous cell carcinoma of the uterine cervix in phase I / II clinical trial. Between May 2006 and January 2012, 26 patients of Protocol 0508 were treated with C-ion RT. The numbers of patients with stage IIB, IIIB, and IVA disease were 13, 11, and 2, respectively. Twenty patients had pelvic lymph node metastases. Median tumor size was 6.1 cm (range, 4.0–10.0 cm). The treatment consisted of extended-field irradiation of 39.0 gray equivalents (GyE) in 13 fractions, and additional 15.0 GyE in 5 fractions was given to the gross tumor volume (GTV) and surrounding tissues. With regard to local boost, 18.0 GyE in 2 fractions was given to GTV only. Total dose to the cervical tumor was 72.0 GyE over 20 fractions. The median follow-up period was 37 months. Twenty-one patients had grade 1 or 2 acute gastrointestinal toxicity, but all patients completed the treatment on schedule. There were no grade 3 or higher late complications, with 8 patients having grade 1 or 2 toxicities, 1 had grade 2 gastrointestinal toxicity and 2 had grade 2 genitourinary toxicity. Four patients (15.4%) developed local recurrence, and 8 patients (30.8%) had distant metastases. The 2-year local control rate, progression-free survival rate and overall survival rate were 83.6%, 61.5% and 73.1%, respectively. There were no severe acute or late complications in this trial. Prophylactic extended-field C-ion RT for locally advanced squamous cell carcinoma of the uterine cervix was a safe treatment. Although the number of patients in this study was small, the results support further investigations to confirm the therapeutic efficacy and to avoid or reduce toxicity.

**Trial Registration:**

UMIN-CTR UMIN000016169

## Introduction

Para-aortic lymph node (PALN) involvement occurs in a significant portion of patients with uterine cervical cancer and increases in frequency with advanced stage. The prevalence of PALN metastasis in locally advanced cervical cancer is 10–40% [1–3]. Several researchers also demonstrated the benefits of prophylactic extended-field irradiation (EFRT) for patients with high-risk cervical cancer [4, 5]. However, pelvic radiation therapy with concurrent chemotherapy improved survival among patients with locally advanced cervical cancer compared with patients treated with EFRT without a concurrent chemotherapy, as the local control rate of pelvic RT with concurrent chemotherapy arm was significantly higher than that of EFRT arms in RTOG 90–01 [6]. Since then, concurrent chemoradiation therapy (CCRT) has become the standard treatment for locally advanced cervical cancer.

Although CCRT had shown improvement of local control and overall survival against RT alone, around 10–15% of patients developed PALN metastasis after CCRT [6–8]. Therefore, several clinical trials conducted EFRT combined with concurrent chemotherapy, but those trials showed an unacceptably high rate of both acute and late severe toxicities [9–11]. In those trials, EFRT targeting PALN was delivered by conventional technique, and thus another strategy was needed for improved tumor control by prophylactic EFRT in patients with high-risk cervical cancer.

In 1994, clinical studies of carbon-ion RT (C-ion RT) were started at the National Institute of Radiological Sciences (NIRS) [12]. Carbon-ion beams possess improved dose localization properties, and this potentiality can produce great effects on tumors while minimizing normal tissue damage. Moreover, they possess a biological advantage due to their high relative biological effectiveness in the Bragg Peak. Several reports have demonstrated the favorable results of C-ion RT in the treatment of malignant tumors. In C-ion RT for cervical cancer, 4 clinical trials were reported prior to the present study [13–16]. Taken together, C-ion RT is expected to provide a higher local control rate for locally advanced bulky cervical cancer without concurrent chemotherapy because of the biological advantage of carbon-ion beams. Our previous trial (Protocol 9902) was a dose-escalation study of C-ion RT alone to the whole pelvis, and was designed for bulky tumors. All patients receiving 72.0 GyE, although not many in number, showed local control [15], suggesting that C-ion RT alone has the potential to improve local control for locally advanced bulky cervical cancer with a total dose of 72.0 GyE.

Despite the better local tumor control in previous trials, distant metastases frequently occurred, with 30.6% of patients in 2 trials (9702 and 9902) developing PALN failure, and the 5-year overall survival rate was still unsatisfactory [13, 15]. We therefore conducted another clinical trial of C-ion RT for locally advanced cervical cancer, using extended-field irradiation without concurrent chemotherapy (Protocol 0508). This article describes the results of acute and late complications, and the outcomes of Protocol 0508.

## Materials and Methods

### Patient eligibility and endpoint

**Fig 1:**
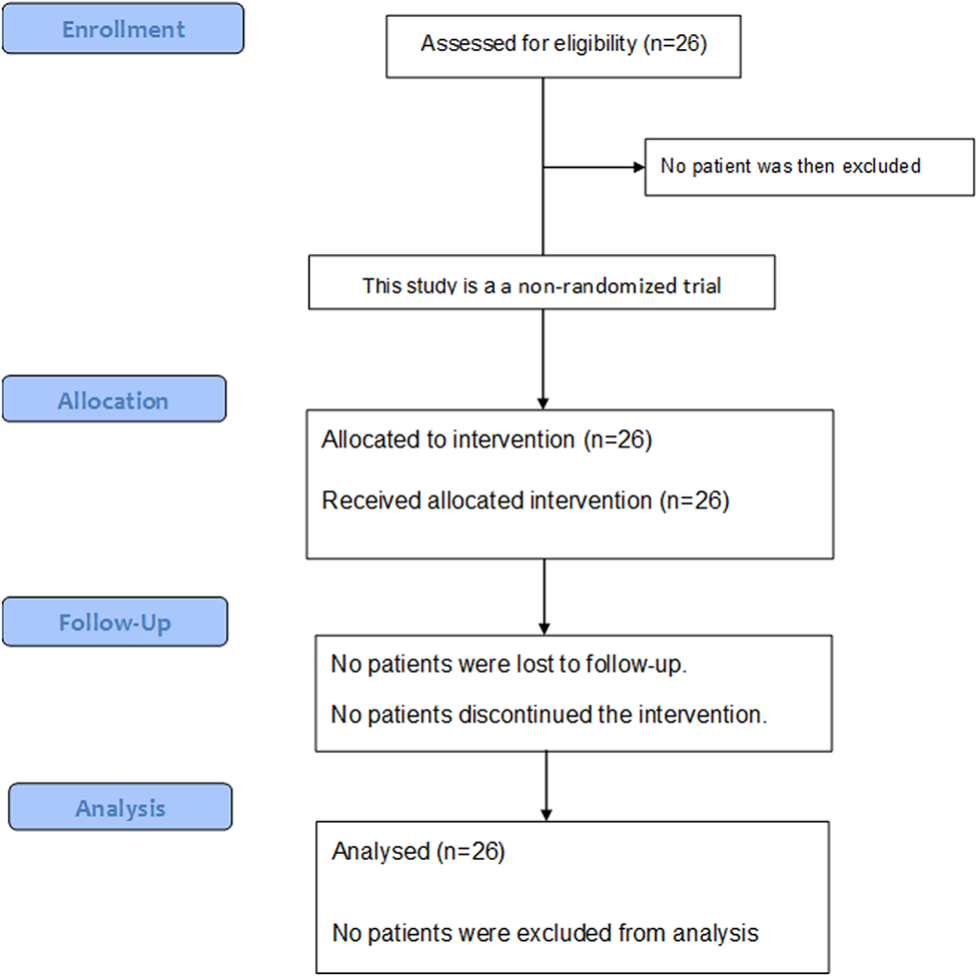
Study Design.

The recruitment and the treatment for this trial were restricted to NIRS. Patients were enrolled into the study if they had previously untreated squamous cell carcinoma of the uterine cervix with International Federation of Gynecology and Obstetrics (FIGO 1994) Stage IIB, III, or IVA disease with a tumor size ≥ 4 cm in diameter, and without rectal invasion. The tumor had to be grossly measurable. Other eligibility criteria included World Health Organization performance status < 3, age ≤ 80 years, and estimated life expectancy of ≥ 6 months. Patients with histories of prior chemotherapy or radiotherapy to the region of the pelvic or para aortic region were excluded from the study. Patients were also excluded if they had severe pelvic infection, severe psychological illness, or active synchronous cancer. Pretreatment evaluation consisted of an assessment of the patient’s history, physical and pelvic examinations by gynecologists and radiation oncologists, cervical biopsy, routine blood cell counts, chemistry profile, chest X-ray, cystoscopy, and rectoscopy. Bladder or rectal involvement was assessed by the findings of endoscopy. Computed tomography (CT) scans of the abdomen and pelvis, magnetic resonance imaging (MRI) of the pelvis, and ^11^C methionine positron emission tomography (PET) scans were also performed for all patients. Patients were staged according to the FIGO staging system, but patients with para-aortic lymph nodes ≥ 1 cm in minimum diameter on CT images were excluded from the studies, although patients with enlarged pelvic lymph nodes only were included. Tumor size was assessed by both pelvic examination and MRI, and dimensions of the cervical tumor were measured according to T2-weighted MRI images. Carbon-11 methionine PET scans were supplementally used for detecting distant metastases. Working group pathologists reviewed the tumor specimens. The treatment protocol for the current study was reviewed and approved by the NIRS Ethics Committee of Human Clinical Research, and all patients provide their written informed consent to participate in this study before the initiation of therapy. The date range for participant recruitment was between May 2006 and January 2012. This study approved by the NIRS Ethics Committee of Human Clinical Research in March of 2006, but Japanese WHO Primary Registry was still not established at that time. So, this clinical trial was registered after the end of trial. The sample size was determined to be 20 based on to the previous trials, and all patients were checked by The Working Group of the Gynecological Tumor on a semiannual basis (Fig 1).

Acute toxicity was established as the primary endpoint in this clinical trial. Secondary endpoints included late toxicity, 2-year local control rate, 2-year overall survival rate and 2-year progression-free survival rate.

### Carbon-ion radiotherapy

The treatment system of C-ion RT has previously been described [17, 18]. Patients were positioned in customized cradles and immobilized with a low-temperature thermoplastic sheet. Treatment planning was based on a set of 5-mm-thick CT images. Three-dimensional treatment planning was performed using HIPLAN software (NIRS, Chiba, Japan) for the planning of C-ion RT [19].

Carbon-ion RT was given once daily, 4 days per week (Tuesday to Friday). At every treatment session, the patient was positioned on the treatment couch with the immobilization devices, and the patient’s position was verified with a computer-aided, on-line positioning system. To minimize internal target positional uncertainty, 100–150 mL of normal saline was infused into the bladder. Patients were also encouraged to use laxatives, if necessary, to prevent constipation throughout the treatment period. The radiation dose was calculated for the target volume and surrounding normal structures and was expressed in GyE, defined as the carbon physical dose (Gy) multiplied by a relative biologic effectiveness value of 3.0 [17, 20].

**Fig 2:**
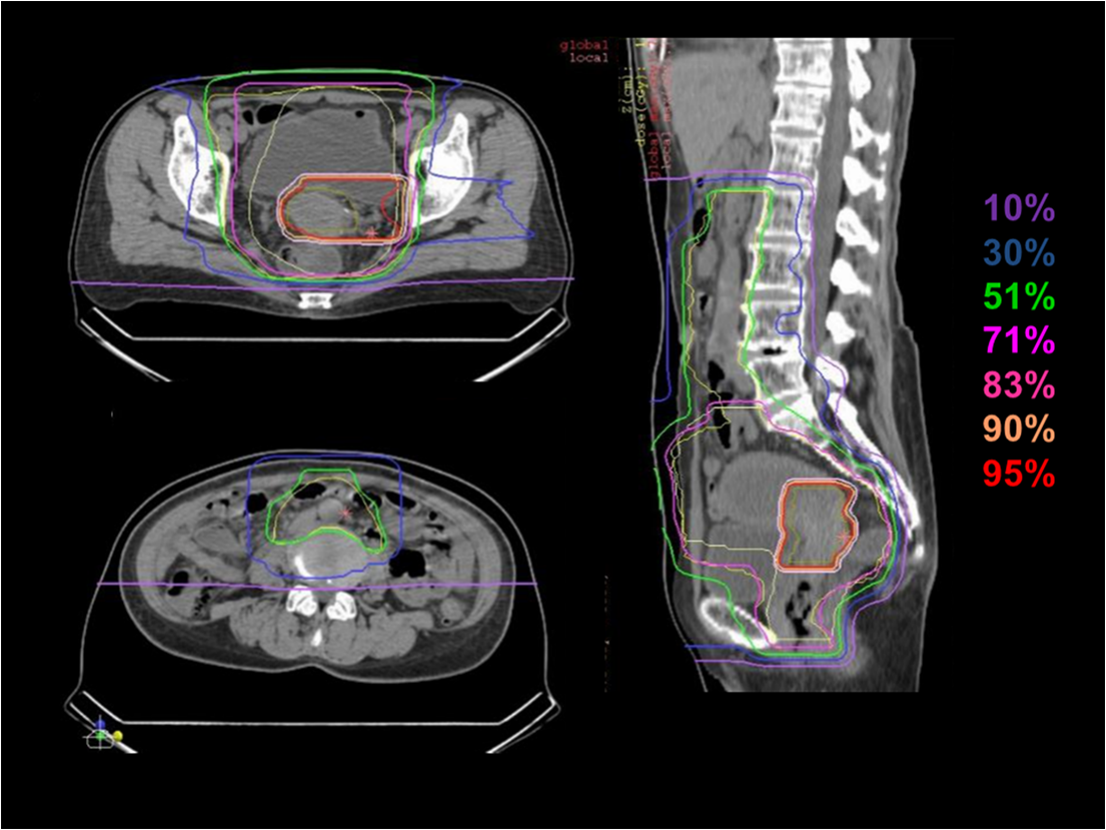
Dose distribution of C-ion RT; Isodose curves of carbon-ion radiotherapy for locally advanced cervical cancer are superimposed on axial and sagittal computed tomography images for the total irradiation plan. Highlighted are 95% (red), 90% (yellow), 83% (orange), 71% (pink), 51% (green), 30% (blue), and 10% (purple) isodose curves.

The treatment consisted of prophylactic extended-field irradiation and local boost. Planning CT scan is basically performed three times during the course of the treatment, and the clinical target volume (CTV) of local boost is reduced twice in accordance with tumor shrinkage. CTV of extended-field irradiation (CTV-1) includes all areas of gross and potentially microscopic disease, consisting of the tumor, uterus, ovaries, parametrium, at least the upper half of the vagina, para-aortic lymph nodes and pelvic lymph nodes (common iliac, internal iliac, external iliac, obturator and presacral lymph nodes), with a superior field border at the space between L1 and L2. The planning target volume-1 (PTV-1) includes CTV-1 plus a 5-mm safety margin for positioning uncertainty. CTV-1 is covered by at least 90% of the prescribed dose. After completing CTV-1 irradiation, first reduction of CTV includes the gross tumor volume (GTV) and uterine cervix, uterine corpus, parametrium, upper half of the vagina, ovaries and swelling lymph nodes (= CTV-2). A 5-mm margin was added to PTV-2. Finally, CTV is shrunk to GTV only (CTV-3), and no margin was added to PTV-3 (Fig 2). Normal tissue structures, such as rectum, sigmoid colon, bladder, and small bowel in the pelvis, were excluded from PTV as much as possible. If PTV-1 and PTV-2 overlapped normal tissues, priority was given to target coverage. However, in the first two clinical trials of C-ion RT (9403 and 9702) for squamous cell carcinoma of the uterine cervix, 18% of patients developed major gastrointestinal (GI) complications [13]. The dose to the GI tracts was limited to < 60 GyE according to DVH analysis, and this limitation had higher priority than the prescription to PTV-3 as final boost irradiation. Based on the results of previous trials, the doses to PTV-1, PTV-2 and PTV-3 were fixed at 39.0 GyE in 13 fractions, 15.0 GyE in 5 fractions and 18 GyE in 2 fractions, respectively, and the total dose to local tumor (GTV) was 72.0 GyE over 20 fractions. All patients did not receive concurrent chemotherapy.

### Assessment of toxicity and efficacy

Patients were followed regularly up to November 2014. After completion of C-ion RT, patients were followed up every 1–3 months for 2 years, and every 3 or 6 months thereafter. All acute toxicity and late toxicity of fracture were graded according to the National Cancer Institute Common Terminology Criteria for Adverse Events, Version 3.0. Late toxicity except fracture was graded according to the Radiation Therapy Oncology Group/European Organization for Research and Treatment of Cancer late radiation morbidity scoring scheme [21]. Treatment effect was evaluated in terms of local control, progression-free survival and overall survival. Local control were defined as no evidence of tumor regrowth or recurrence in the treatment volume according to physical examination, CT, MRI, PET, and/or biopsy. Local control, progression-free survival and overall survival rates were calculated by Kaplan-Meier method, which were performed with SPSS software, version 16.0.

## Results

Twenty-six patients were enrolled into Protocol 0508 between May 2006 and January 2012, no patient was then excluded, and all 26 patients were evaluated in this study. Patient characteristics are summarized in Table 1. Staging laparotomy was not performed, and no histologic confirmation of CT-positive pelvic or para-aortic lymph nodes was obtained. No patient underwent lymph node resection. Overall treatment time (OTT) ranged from 32 to 39 days, with a median of 35 days. The median follow-up durations of all patients and surviving patients were 38 and 49 months, respectively. No patients were lost to follow-up.

**Table 1:** Patient characteristics.

No. of patients		26
Follow-up	range (median) (mo)	8–85 (38)
Age	range (mean) (y)	32–78 (59)
Performance status	0–1	26
Stage (FIGO)	IIB	13
	IIIB	11
	IVA	2
Pelvic lymph node status	Negative	6
	Positive	20
Tumor size	range (cm) (median)	4.0–10.0 (6.1)
	< 5.0	7
	5.0–6.9	13
	≥ 7.0	6

All of the observed acute and late toxicities are listed in Tables 2 and 3. All patients completed the scheduled therapy. No patient developed grade 3 or higher acute non-hematological toxicity and grade 4 or higher acute hematological toxicity. All patients for grade 2 or 3 anemia were caused by genital bleeding from local tumor. Eight patients developed late sigmoid colon or rectum complications. No patients developed grade 3 or higher late GI or GU toxicity. Two of 26 patients developed grade 2 compression fracture of the lumbar spine.

**Table 2:** Acute toxicity.

	(N)	G0	G1	G2	G3	G4
**Upper GI**	**26**	**7**	**14**	**5**	**0**	**0**
**Small intestine**	**26**	**4**	**15**	**6**	**0**	**0**
**Rectum/ sigmoid**	**26**	**24**	**2**	**0**	**0**	**0**
**GU**	**26**	**22**	**3**	**1**	**0**	**0**
**Skin**	**26**	**20**	**6**	**0**	**0**	**0**
**Neutropenia**	**26**	**22**	**1**	**3**	**0**	**0**
**Anemia**	**26**	**5**	**9**	**10**	**2**	**0**
**Thrombopenia**	**26**	**25**	**1**	**0**	**0**	**0**

GI: gastrointestinal toxicity, GU: genitourinary toxicity

**Table 3:** Acute toxicity.

	(N)	G0	G1	G2	G3	G4
**Upper GI**	**26**	**7**	**14**	**5**	**0**	**0**
**Small intestine**	**26**	**4**	**15**	**6**	**0**	**0**
**Rectum/ sigmoid**	**26**	**24**	**2**	**0**	**0**	**0**
**GU**	**26**	**22**	**3**	**1**	**0**	**0**
**Skin**	**26**	**20**	**6**	**0**	**0**	**0**
**Neutropenia**	**26**	**22**	**1**	**3**	**0**	**0**
**Anemia**	**26**	**5**	**9**	**10**	**2**	**0**
**Thrombopenia**	**26**	**25**	**1**	**0**	**0**	**0**

GI: gastrointestinal toxicity, GU: genitourinary toxicity

**Fig 3:**
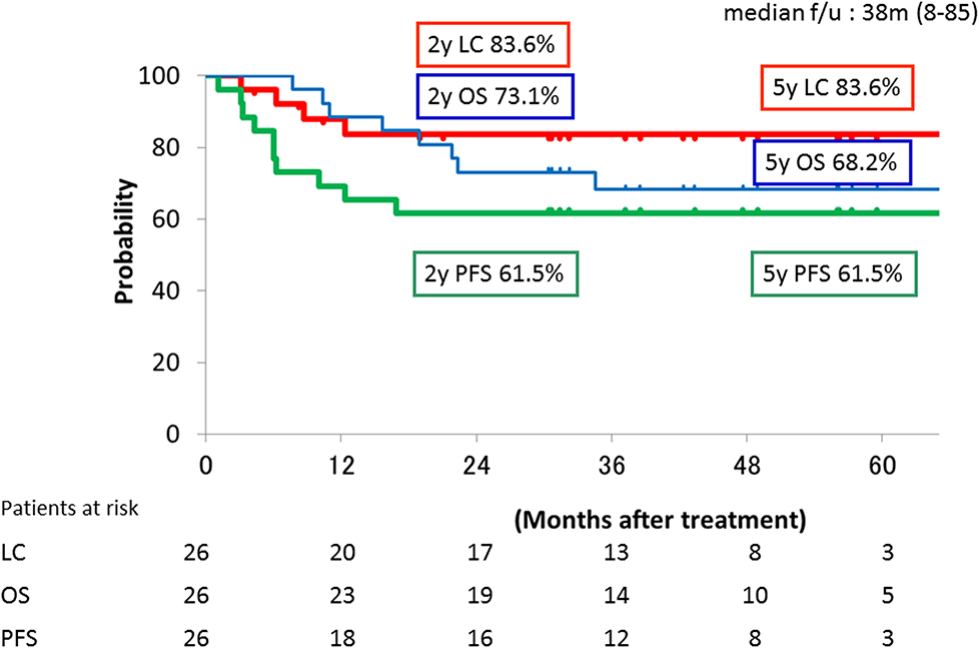
The local control, progression-free survival and overall survival curves; Local control (red line), progression-free survival (green line), and overall survival curves (blue line) are shown for all patients treated with carbon-ion radiotherapy.

Fig 3 shows the overall survival rate, local control rate and progression-free survival rate. The 2-year and 5-year local control rates were both 83.6%. The 2-year and 5-year overall survival rates were 73.1 and 68.3%, respectively. The 2-year and 5-year progression-free survival rate were both 61.5%. Four patients (15.4%) developed local failure, 3 patients (11.5%) had pelvic lymph node failure, and 8 patients (30.8%) had distant failure. One of the 8 patients with distant failure showed PALN failure only, and the other 7 had distant failure without PALN failure. Two of the 3 patients with pelvic lymph node failure had systemic failure (local, regional and distant failure). Cumulative 2-year and 5-year PALN failure rates were both 5.3% (95%CI: 0–15.3%).

## Discussion

Protocol 0508 was the fourth clinical trial of C-ion RT for locally advanced squamous cell carcinoma of the uterine cervix. The treatment consisted of prophylactic extended-field irradiation of 39.0 GyE in 13 fractions, GTV and surrounding tissues of 15.0 GyE in 5 fractions, and local boost of 18.0 GyE in 2 fractions to the cervical tumor. The total dose to local tumor was 72.0 GyE over 20 fractions. All patients were at high risk for PALN failure because they had bulky tumor or/and pelvic lymph node metastases. This trial revealed that prophylactic extended-field C-ion RT with local boost achieved safe treatment, better local control, and reduction of PALN failure.

This clinical trial revealed extended-field C-ion RT with local boost to be a safe treatment for locally advanced cervical cancer. The distant metastasis rate is very high in patients with locally advanced and bulky tumors, and especially PALN recurrence is a major problem of distant metastases [1–3]. Nelson et al. reported that PALN metastasis occurs in 14.9% and 38.4% of stage IIB and stage IIIB patients, respectively, based on PALN biopsies [1]. Therefore, EFRT combined with concurrent chemotherapy is expected to become one of the curable strategies. RTOG 0116 conducted EFRT combined with cisplatin chemotherapy for cervical cancer [10]. Small et al. reported the results of this combined treatment, but concluded that it was associated with high rates of acute and late toxicities. They showed that 84% of patients developed grade 3 or higher acute toxicities and 40% had grade 3 or higher late toxicities [10]. The current study, which was extended-field C-ion RT without concurrent chemotherapy, showed that there were no grade 3 or higher acute and late toxicities (Tables 2, 3) even though a higher local control rate was maintained and shorter OTT was achieved (Table 4). These results were achieved on the basis of better dose distribution of C-ion RT compared with that of photon irradiation because of the physical aspects of C-ion beams (Fig 2). Thus, extended-field C-ion RT can be considered a safe treatment for locally advanced cervical cancer.

**Table 4:** Comparison of studies of extended-field radiation therapy.

Study or ref.	Year of publication	No. of patients	OTT (median)	RT technique	Chemotherapy	Acute GI toxicity (≥G3) (%)	Late GI toxicity (≥G3) (%)	5y Locoregional control rate (%)
**Eifel et al. (RTOG 90–01)**	**2004**	**195**	**58 days**	**Conv.**	**None**	**1.6**	**9.3% (large bowel and rectum)**	**66**
**Grigsby et al. (RTOG 92–10)**	**2001**	**30**	**54 days**	**Conv.**	**Concurrent CDDP +5FU**	**56**	**21**	**50**
**Small et al. (RTOG0116)**	**2007**	**26**	**NA**	**Conv.**	**CDDP**	**35**	**27**	**62 (15m)**
**Jensen et al.**	**2013**	**21**	**69 days**	**IMRT**	**CDDP**	**19**	**0**	**90.5 (18m)**
**Beriwal et al.**	**2007**	**36**	**56 days**	**IMRT**	**CDDP**	**3**	**3**	**80 (2y)**
**Poorvu et al.**	**2013**	**46**	**NA**	**IMRT**	**Concurrent or sequential**	**6.5**	**6.5**	
**Current study**		**26**	**35 days**	**C-ion RT**	**none**	**0**	**0**	**83.6 (2y)**

OTT: overall treatment time; GI: gastrointestinal; RT: radiation therapy; CDDP: cisplatin; 5FU: 5-fluorouracil IMRT: Intensity Modulated Radiation Therapy; Conv.: Conventional radiation therapy

**Fig 4:**
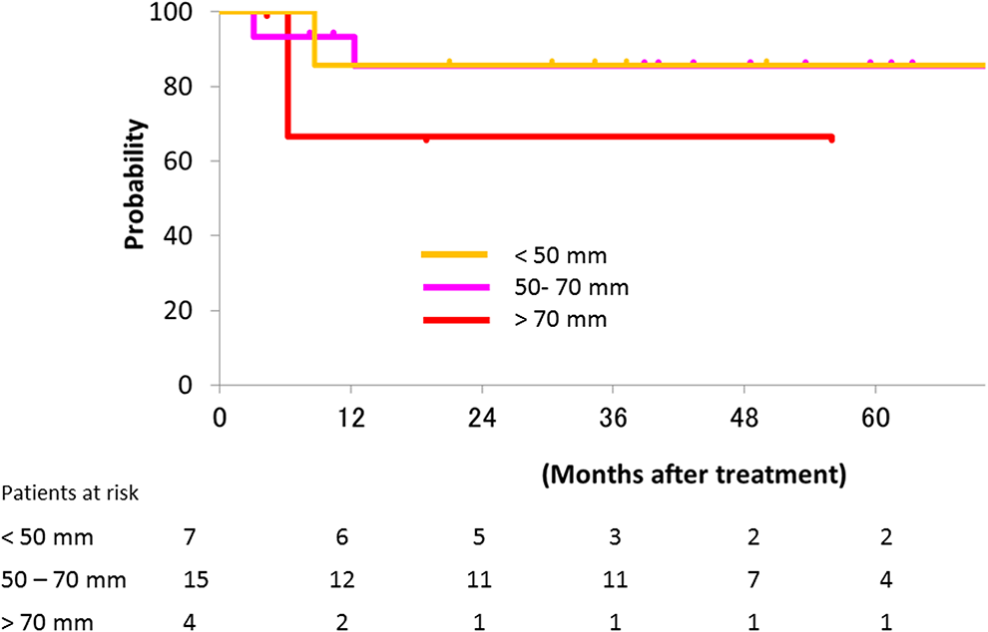
The local control in patients with tumors < 50 mm, 50–70 mm, and > 70 mm.

C-ion RT achieved better local control for bulky cervical cancer in this trial. Our previous trials suggested that C-ion RT has the potential to improve local control for locally advanced bulky cervical cancer with a total dose of 72.0 GyE [13, 15]. Therefore, this trial used 72.0 GyE over 20 fractions to the local tumor. Toita et al. reported 2-year locoregional control rates for CCRT in patients with tumors < 50 mm, 50–70 mm, and > 70 mm of 85%, 72%, and 54%, respectively [7]. Parker et al. reported 5-year local control rates for < 50 mm and > 50 mm of 73% and 56%, respectively [22]. The 2-year local control rate in the current study was 83.6% despite the median tumor size of our patients being 6.1 cm (4–10 cm). The number of patients with tumors < 50 mm, 50–70 mm, and > 70 mm were 7, 15 and 4, respectively. The both 2-year and 5-year local control rate for < 50 mm, 50–70 mm, and > 70 mm were 86%, 86%, and 67%, respectively (Fig 4). Thus, C-ion RT has the potential to improve local control for advanced bulky cervical cancer without concurrent chemotherapy.

Prophylactic extended-field C-ion RT reduced PALN failure in this trial. Distant metastases, especially PALN metastases, occur at higher frequency in patients with bulky tumors. Toita et al. reported a 2-year distant failure rate of 19% for tumors < 50 mm, 20% for 50–70 mm, and 47% for > 70 mm, concluding that the incidence of distant failure increased with larger tumor size as well [7]. In two previous studies of C-ion RT (Protocols 9702 and 9902), the PALN failure rate was 25.0% [23]. Especially in pelvic lymph node-positive patients, PALN failure was seen at a high rate of 44.4%. In the current study, 1 of 26 patients developed PALN failure after extended-field C-ion RT and cumulative PALN failure rate was 5.3% (95%CI: 0–15.3%), even though 20 of the 26 patients had pelvic lymph node metastases before treatment initiation. Hence, 39.0 GyE over 13 fractions of prophylactic C-ion RT for the PALN region will improve regional control including PALN in locally advanced cervical cancer.

Previously, prophylactic irradiation to PALN regions was performed with high expectations of survival benefits, and several trials were conducted for patients with high-risk cervical cancer. However, CCRT provided clinical benefit against EFRT alone, as CCRT had higher local control [6]. Furthermore, EFRT combined with concurrent chemotherapy indicated intolerable treatment [10]. Therefore, the clinical benefits of prophylactic irradiation to PALN regions for patients with high-risk cervical cancer still remain unclear. Recently, several clinical trials reported that whole pelvic intensity modulated radiation therapy (IMRT) achieved lower GI and GU acute toxicities than whole pelvic conventional radiation therapy [24, 25], so extended-field IMRT (EF-IMRT) combined with concurrent chemotherapy expected the improvement of therapeutic efficacy and toxicity. Several researchers reported EF-IMRT combined with concurrent chemotherapy, and they showed less acute and late toxicities (Table 4) [26, 27]. Thus, by the use of new technologies such as C-ion RT or EF-IMRT with chemotherapy, which should achieve higher local control and lower toxicities, re-evaluation of prophylactic irradiation to PALN regions can be expected to demonstrate clinical benefits.

Nevertheless, the 2-year and 5-year overall survival rates in this study were 73.1% and 68.2%, respectively, which are still unsatisfactory. This was because 26.9% of the patients developed distant failure exclusive of PALN failure, in spite of the fact that PALN failure was reduced by prophylactic PALN irradiation. This clinical trial did not include concurrent chemotherapy, as the effect of C-ion RT combined with chemotherapy at that time had not been known. Thus, to improve the survival rates, with the understanding that distant metastases need to be reduced, we are now conducting a new clinical trial of C-ion RT combined with chemotherapy. Whereas, 2 of 26 patients developed grade 2 compression fracture of the lumbar spine. They were older than 70 years old, and the lumbar spine received relatively lower doses (< 20GyE) for C-ion RT. However, these fractures could not deny the possibility of treatment related toxicity according to the discussion on The Working Group of the Gynecological Tumor. Thus these need to be careful observation continually.

In conclusion, prophylactic extended-field C-ion RT for locally advanced squamous cell carcinoma of the uterine cervix achieved safe treatment, better local control and reduction of PALN failure. Although the number of patients in this study was small, the results support the need for further investigations to confirm the therapeutic efficacy and toxicity. In the future, although re-evaluation of the clinical benefit of prophylactic irradiation to PALN regions by C-ion RT or other new techniques will have high expectations.

## Supporting Information

S1 TREND Checklist TREND Statement Checklist. (PDF)

S1 Protocol Protocol of this clinical trial in Japanese. (DOC)

S2 Protocol Translated Protocol of this clinical trial. (DOCX)
