# Correction: Clinical Trial of Prophylactic Extended-Field Carbon-Ion Radiotherapy for Locally Advanced Uterine Cervical Cancer (Protocol 0508)

**DOI:** 10.1371/journal.pone.0143301

**Published:** 2015-11-13

**Authors:** Masaru Wakatsuki, Shingo Kato, Hiroki Kiyohara, Tatsuya Ohno, Kumiko Karasawa, Tomoaki Tamaki, Ken Ando, Hirohiko Tsujii, Takashi Nakano, Tadashi Kamada, Makio Shozu, The Working Group of the Gynecological Tumor

There is an error in Table 3. Please see the corrected Table 3 here:

**Table 3:** Late toxicity

	(N)	G0	G1	G2	G3	G4
**Upper GI**	**26**	**23**	**3**	**0**	**0**	**0**
**Small intestine**	**26**	**24**	**2**	**0**	**0**	**0**
**Rectum/ sigmoid**	**26**	**18**	**7**	**1**	**0**	**0**
**GU**	**26**	**21**	**2**	**3**	**0**	**0**
**Fracture**	**26**	**16**	**8**	**2**	**0**	**0**
**Skin**	**26**	**26**	**0**	**0**	**0**	**0**

GI: gastrointestinal toxicity, GU: genitourinary toxicity
