# Supplementary material for: Clinical Trial of Prophylactic Extended-Field Carbon-Ion Radiotherapy for Locally Advanced Uterine Cervical Cancer (Protocol 0508)
Source: PLoS One. 2015 May 20;10(5):e0127587. doi: 10.1371/journal.pone.0127587 (PMC4439043; doi:10.1371/journal.pone.0127587)
Supplement: S1 Protocol — (DOC) [file pone.0127587.s002.doc]

放射線医学総合研究所

重粒子線治療ネットワーク会議／計画部会

婦人科分科会

局所進行子宮頚部扁平上皮癌に対する炭素イオン線による骨盤および傍大動脈リンパ節領域への

同時予防照射に関する第I/II相試験計画書

（略称:子宮IV）

A phase I/II clinical trial of prophylactic extended-field radiotherapy of pelvic and para-aortic lymph nodes in locally advanced squamous cell carcinoma of the uterine cervix using carbon-ion beams.

研究代表者

鈴木通也

研究事務局

加藤真吾

放射線医学総合研究所重粒子医科学センター

千葉市稲毛区穴川4-9-1

計画書案

初稿　2005年11月24日

第2稿　2006年1月7日

第3稿　2006年2月15日

目次

1　試験の概要

1-1　目的

1-2　試験の種類

1-3　対象

1-4　試験治療の内容

1-5　安全性／有効性の評価指標

1-6　試験期間と予定症例数

2　臨床試験の構成

2-1　臨床試験の構成の模式図

2-2　臨床試験の構成の説明

3　背景

3-1　局所進行子宮頚癌の治療の現状と重粒子線治療について

3-2　これまでの臨床試験の経緯

3-3　傍大動脈リンパ節に対する予防照射の合理的根拠

4　目的と評価指標

　4-1　試験の目的

4-2　評価指標

5 対象の選択

5-1　適格条件

5-2　不適格条件

6　インフォームド・コンセントと倫理審査

6-1　インフォームド・コンセント

6-2　同意の取得

6-3　倫理審査および承認

7　登録と追跡

　7-1　登録

7-2　追跡調査および調査期間

8　試験期間と予定症例数

8-1　予定症例数

8-2　試験期間

8-3　試験期間の延長

９ 炭素イオン線治療

9-1　使用機器

9-2　標的体積

9-3　治療計画

9-4　炭素イオン線と照射野の整形

9-5　位置決め法

9-6　線量指示、線量分割、照射法

9-7　治療内容

9-8　線量の制限

9-9　治療中、治療後の他の治療の併用について

10　病理

　10-1　初回の病理標本

　10-2　治療後の病理標本

　10-3　剖検

11　必要な評価項目および検査

11-1　治療開始前検査

11-2　治療期間中および観察期間、追跡期間中の評価・検査

11-3　腫瘍縮小効果判定のための検査

12　予期される有害反応と対策、治療変更基準

12-1　予期される有害反応

12-2　炭素イオン線治療の変更・中止基準

12-3　有害反応／有害事象に対するその他の対処方法

12-4　予期できない有害反応／有害事象に対する対処方法

13　炭素イオン線治療終了後の治療

14　定期的な臨床試験の評価と報告

14-1　目的、時期、結果の報告

14-2　臨床試験の評価項目

14-3　プロトコール運用委員会

15　登録の終了と中止およびプロトコール改訂

15-1　症例登録の終了

15-2　試験の中止

15-3　プロトコール改訂

16　報告義務のある有害反応／有害事象

16-1　有害反応／有害事象の報告義務

16-2　担当医の報告義務

16-3　研究事務局／プロトコール運用委員会の義務

17　解析関連事項

17-1　評価指標の定義と解析方法

17-2　解析時の症例の取り扱い

18　研究組織

18-1　研究代表者

18-2　研究事務局

19　参考文献

資料１.患者の同意・承諾書 （同意説明文書）

資料２.患者の適格性を示す文書（適格性確認表）

資料３.患者の主訴、既往歴、現病歴などを示す文書（患者病歴）

資料４.研究登録書

資料５.臨床医学研究倫理審査放射線治療部会審査判定書

資料６.臨床医学研究倫理審査放射線治療部会審査判定通知書

資料７.重粒子線治療婦人科臨床研究班

資料８.重粒子線治療計画部会婦人科分科会

資料９.重粒子線治療ネットワーク会議評価部会

資料１０.重粒子線治療ネットワーク会議計画部会

資料１１.重粒子線治療ネットワーク会議

資料１２.放射線医学総合研究所臨床医学研究倫理審査放射線治療部会

資料１３.放射線医学総合研究所臨床医学研究倫理審査委員会

資料１４. RTOG Acute Radiation Morbidity Scoring System

資料１５. RTOG/EORTC Late Radiation Morbidity Scoring System

資料１６.炭素イオン線AE/AR急送一次報告書

資料１７.炭素イオン線AE/AR報告書

　（資料５～１７：１１．別添資料参照）

１　試験の概要

１－１　目的

局所進行子宮頸部扁平上皮癌症例を対象に、骨盤および腹部傍大動脈リンパ節領域を併せた拡大照射野に対する炭素イオン線による予防照射療法の安全性および有効性を確認する。

１－２　試験の種類

　　臨床第I/II相試験

１－３　対象

1-3-1適格条件

1. 生検（組織診）で証明された子宮頸部扁平上皮癌。

2. FIGOの臨床病期のIIB期で最大腫瘍径が4cm以上のもの、ないしIIIB期、IVA期。

3. 腹部CTにて腹部傍大動脈領域に短径1cm以上のリンパ節が描出されない。

4. 計測可能な病変である。

5. 年齢は80歳以下である。

6. PSは0～2である。

7. 十分な骨髄機能を有する。

白血球 > 3000/mm^3^

ヘモグロビン > 10g/dl

血小板 > 100,000/mm^3^

8. 6ヶ月以上の生存が見込まれる。

9. 文書による同意が得られている。

1-3-2 不適格条件

1. 重篤な合併症（例えば制御困難な心疾患・高血圧・糖尿病、難治性の感染症、急性期の消化性潰瘍、制御困難な精神病など）を有する。

2. 活動性の重複癌を有する。

3. 当該照射部位に放射線治療の既往がある。

4. 化学療法の既往がある。

5. 医学的、心理学的または他の要因により担当医師が不適当と考える。

１－４　試験治療の内容

炭素イオン線治療は放射線医学総合研究所重粒子医科学センターに設置された医用重粒子加速器（HIMAC）および照射装置を用いる。骨盤部への治療は、まず子宮頸部の病巣から骨盤リンパ節まで含む領域に対して、1日1回3.0 GyE、2週間で6-8回を原則として、合計39.0 GyEの照射を行う（骨盤照射）。骨盤照射の上縁は総腸骨動静脈分岐部付近の高さとする。次いで照射範囲を子宮頸部の病巣から腫瘍浸潤の可能性が高い子宮傍結合織・子宮体部・膣上部などに絞って、1日1回3.0 GyEで5回、合計15.0 GyEの照射を行う。最後に治療範囲を肉眼的腫瘍体積に絞り、消化管を完全に照射範囲から外して、1日1回9.0 GyEで2回、合計18.0 GyEの照射を行う。全分割回数は20回、全治療期間は5週間で、肉眼的腫瘍体積に対する総線量は72.0 GyEとなる。

傍大動脈リンパ節に対する治療は骨盤照射と同日に行う。傍大動脈リンパ節の治療範囲の上縁はL1の高さとし、下縁は骨盤照射の治療範囲の上縁と接するようにして、本領域に1日1回3.0 GyE、2週間で6-8回を原則として、合計39.0 GyEの照射を行う。

**治療方法概要**

　治療期間5週間20回照射法

　　 １．骨盤リンパ節および腹部傍大動脈リンパ節領域を含む拡大照射野

　　　　　カーボン　　3.0 GyE　13回　約3週間　　 39 GyE

　　 ２．骨盤内で腫瘍浸潤を考慮したやや広めの局所照射野

　　　　　カーボン　　3.0 GyE 5回　 約1週間　　 15 GyE　　総線量　54 GyE

　　 ３．腸管を避け子宮頸部の肉眼的腫瘍体積のみに限局した照射野

　　　　　カーボン　　9.0 GyE　2回　 2日　　　　 18 GyE　 総線量　72 GyE


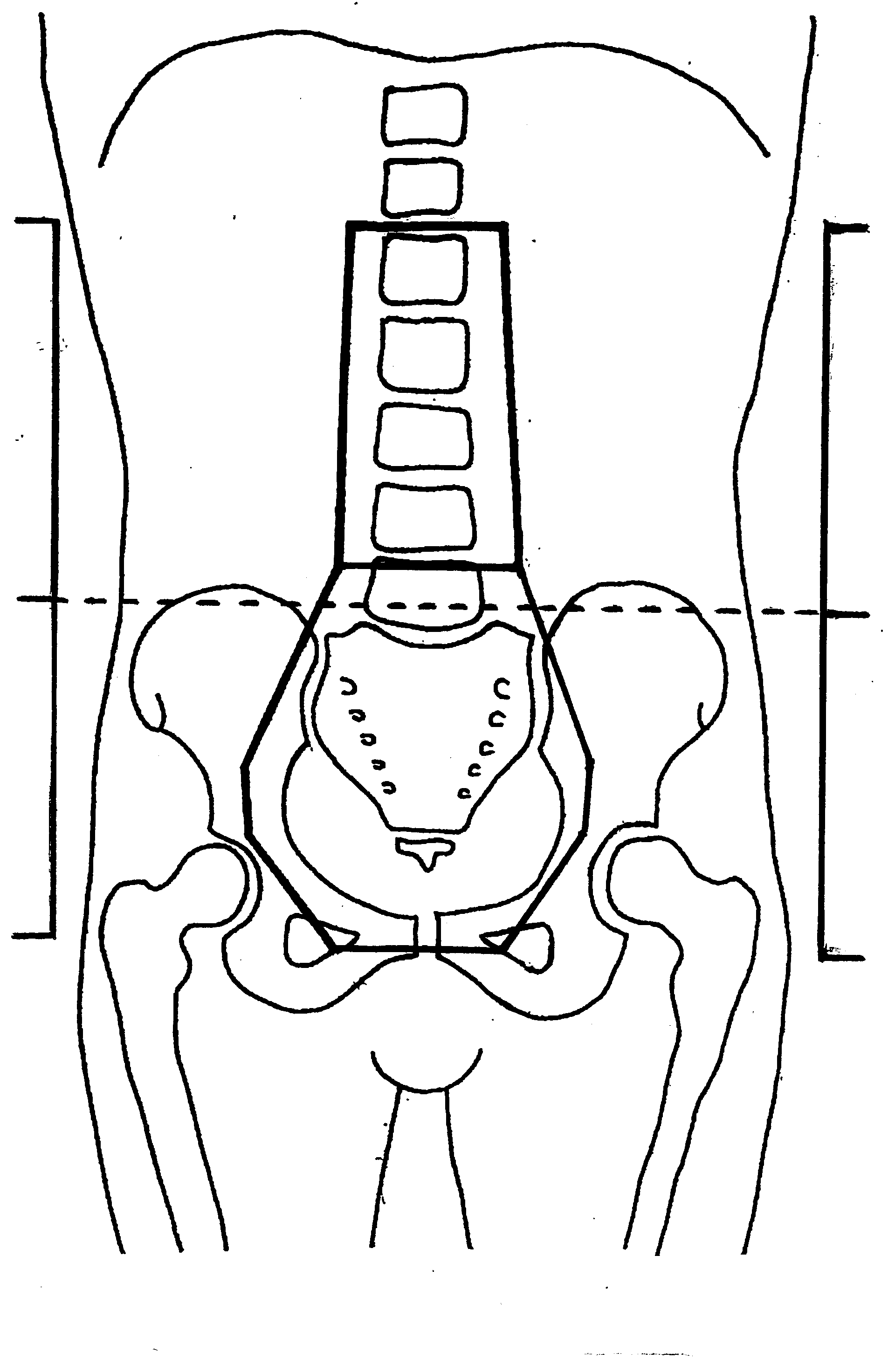


＊骨盤照射と傍大動脈リンパ節領域の照射は、前方・左方・

右方からの３門で行い、両方の照射は同一日には同一方向

から行う。

前方からの照射野のつなぎ目はほぼL5上縁の高さ、左右

からの照射野のつなぎ目はほぼL5/S1の高さとし、両者の

間隔は原則として2 cmとする。つなぎ目の部分の線量は

予定線量の+5%を超えない範囲とする。

＊骨盤照射と傍大動脈リンパ節領域の照射は、原則として

呼吸同期照射とする。

＊第2，第3段階ともCTを撮り治療計画を行う。

　骨盤リンパ節＋傍大動脈リンパ節領域 子宮頸部の肉眼的腫瘍体積に

を含む拡大照射　　　　　 限局した照射

やや広めの局所照射

１－５安全性／有効性の評価指標（エンドポイント）

1-5-1主要評価指標（プライマリーエンドポイント）

　　1) 正常組織の早期反応

1-5-2副次的評価指標（セカンダリーエンドポイント）

　 1) 正常組織の遅発性反応

2) 局所制御期間

　　3) 無再発生存期間

１－６試験期間と予定症例数

　１）登録期間は2006年4月－2008年3月まで2年間、追跡調査期間は登録終了後2年間とする。

２）登録症例数は20例を目標とする。

２　臨床試験の構成（スタディーデザイン）

2-1臨床試験の構成図（図1)

　2-2臨床試験の構成の説明

- 放射線医学総合研究所婦人科腫瘍担当医師（以下担当医師）は患者の適格性を確認する。問題のある場合には婦人科腫瘍プロトコール運用委員会に症例を提示し、患者の適格性の再確認を行う。
- 適格と判断された場合、担当医師は、試験内容を説明し文書による同意を本人から得る。
- 同意が得られた後、担当医は３次元治療計画を行い、標的体積を決定すると同時に照射体積に含まれる正常臓器の線量を計算し、照射線量、照射方向等を決定し、結果を炭素イオン線治療カンファレンスに提示し、指示線量および線量分布等につき再確認を行う。
- 担当医師は、定められた様式の適格性確認表、患者病歴に記載し、説明同意文書と共に放射線医学総合研究所臨床医学研究倫理審査放射線治療部会に提出し、承認を受ける。
- 担当医師は臨床医学研究倫理審査放射線治療部会審査判定通知書により承認を確認した後、炭素イオン線治療を開始する。
- 担当医師は炭素イオン線治療終了後、腫瘍縮小効果、局所制御につき『炭素イオン線治療の評価指針』（平成13年2月版）に従い判定する。
- 担当医師は照射に伴う急性期および晩期の有害反応について『炭素イオン線治療の評価指針』（平成13年2月版）にしたがい判定する。
- 担当医師は、プロトコール運用委員会の指示に従い、線量の増減を行う。
- 担当医師は、局所再発あるいは転移が認められた場合、第１３項炭素イオン線治療終了後の治療に従い適切な処置を行う。
- 担当医師は、有害反応が認められた場合、第１２項予期される有害反応と対策、治療変更基準に従い適切な処置を行う。
- 担当医師は定期的に腫瘍効果ならびに正常組織の反応の観察に必要な画像診断等の検査の指示を行う。

臨床試験の構成図（図１）


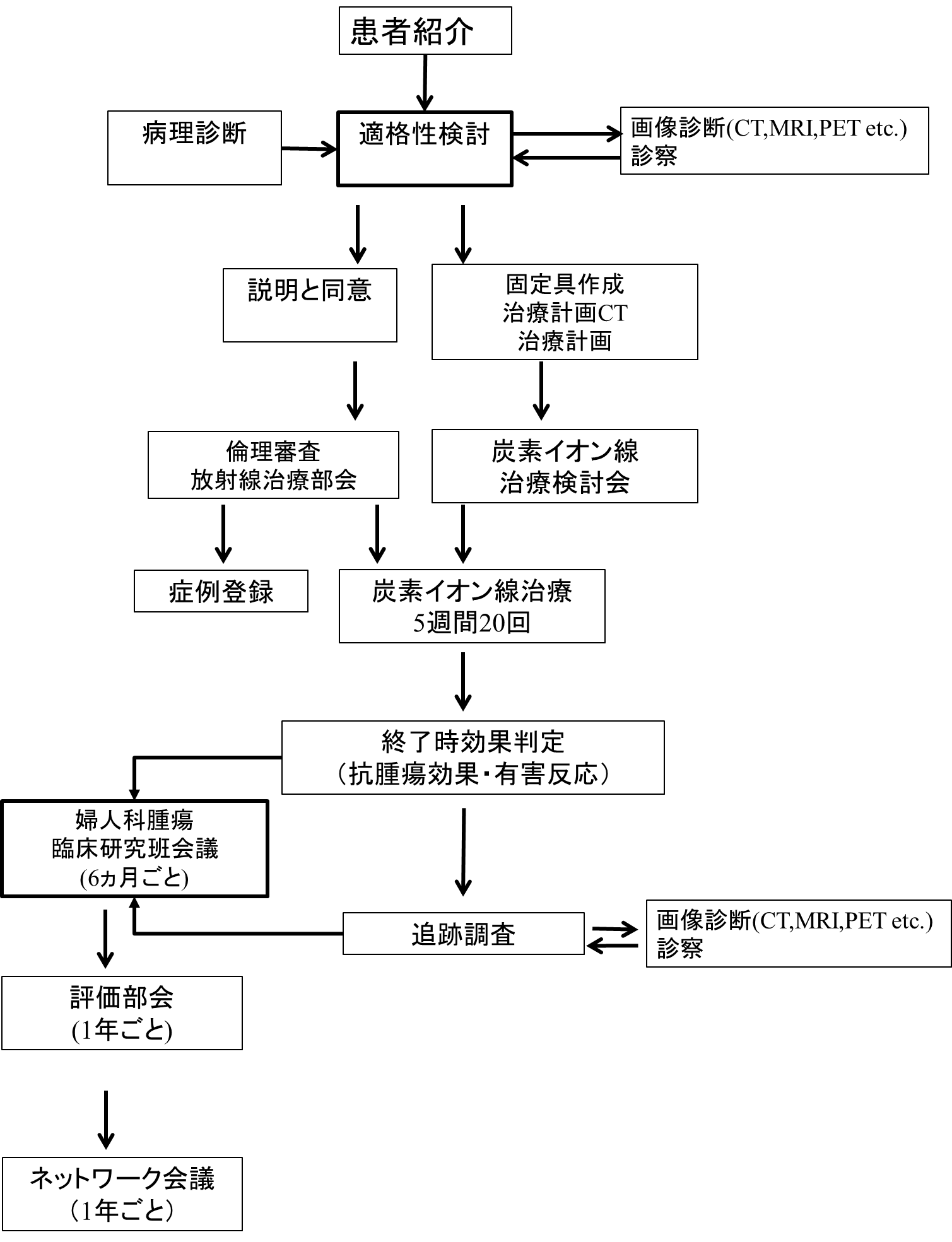


３　背景

- 1. 局所進行子宮頚癌の治療の現状と重粒子線治療について

局所進行子宮頸癌に対する治療においては，シスプラチンを主体とした化学療法と放射線治療の同時併用療法（化学放射線治療）と従来の放射線単独治療とのランダム化比較試験が、1990年代に米国を中心に行われた。それらの第III相臨床試験および他の臨床試験を合わせたメタ解析の結果、化学放射線治療は放射線単独治療に比して、局所制御率および全生存率を明らかに向上させることが報告された。この結果を受けて、現在では化学放射線治療が局所進行子宮頸癌に対する標準治療となってきている（文献1-4）。ただし本治療法においても腫瘍の大きさや浸潤程度の増加とともに治療成績は不良となり、臨床病期III-IVA期（IVA期の割合10%）の5年局所制御率および全生存率はそれぞれ約70%、60%である（文献1）。子宮頸癌に対する腔内照射では、腫瘍に高線量を照射しかつ周囲の正常組織への照射線量を低減することができ、腫瘍の局所制御に重要な役割を果たしている。しかしながらIII-IVA期の大きな腫瘍においては、通常のタンデムとオボイド線源による腔内照射では腫瘍全体に十分な線量を投与することができず、これが局所制御不良の原因の一つと考えられる（文献5）。

重粒子線の一種である炭素イオン線は、通常の放射線治療に使われている高エネルギーＸ線やγ線とは異なる物理学的および放射線生物学的な特徴を有している。Ｘ線やγ線は人体を透過してしまうのに対して、炭素イオン線の場合は任意の深さで止めることが可能である。さらに炭素イオンは停止直前に線量が最大となるブラッグピーク (Bragg’s peak)を形成すると同時に、標的より深い部位への線量寄与はほとんどない。重粒子線加速器HIMAC では、標的体積の深さに応じて炭素イオン粒子を最適エネルギーに加速し、深部がん治療に適した線量分布を作ることが可能である。さらに、炭素イオン線の場合はブラッグピーク領域では高LET( linear energy transfer )放射線としてＸ線に比べて高い生物効果を呈するため、放射線抵抗性腫瘍に対しても有効であると考えられる。したがって炭素イオン線では、通常の放射線に比べて標的領域に高線量を集中できると同時に、高い生物効果を有するため、通常の放射線治療では難治性の進行子宮頸癌において局所制御の向上が期待できる。

- 1. これまでの臨床試験の経緯

局所進行子宮頸癌に対しては、1995年6月から４つの臨床第I/II相試験（プロトコール9403（子宮I）、9702（子宮II）、9704（子宮腺癌）、9902（子宮III））を行い、本疾患に対する炭素イオン線治療の安全性と有効性を検討してきた。その結果、安全性に関しては、１）子宮頸部から骨盤リンパ節領域を含む広い治療範囲への36.0 GyE/12Fr～48.0 GyE/16Frの照射は、高度の急性反応を発生させることなく安全に施行しうること、２）消化管におけるGrade 3-4の遅発性反応症例の解析から、消化管に対する重篤な遅発性反応の発生を抑えるためには消化管の線量を60.0 GyE未満に抑えるべきであることが明らかとなった。これらの結果に基づいて照射法に改善を加えた結果、現行の2つのプロトコール（9704、9902）で治療し6か月以上経過を観察した全51症例では、消化管の遅発性反応はGrade 1は13例（25%）にみられたが、Grade 2および4はそれぞれ1例に（2%）発生したのみであった（経過観察期間8-85か月、中央値24.6か月）。

一方、有効性に関しては、子宮頸部扁平上皮癌のプロトコール9702と9902の計35症例の解析（経過観察期間8-93か月、中央値27.2か月）から、１）c-N0症例では骨盤リンパ節領域に対する39.0 GyE/13Fr～44.8 GyE/16Frの照射で骨盤リンパ節再発は認められず、リンパ節に対する予防照射の線量は40 GyE前後で十分と考えられること、２）局所制御率に関しては、総線量64.0-68.8 GyEにおいては61%（14/23；経過観察期間7-76か月、中央値24か月）であったのに対して、72.0-72.8 GyEでは92%（11/12；同8-93か月、26か月）と線量の増加とともに改善し、原発巣の制御には72 GyE前後の線量が必要であることが示唆された。

プロトコール9702と9902の35症例全体の5年局所制御率は71%で、IIIB-IVA期（IVA期の割合24%）で腫瘍径中央値6.5cmと大きな腫瘍を対象としていることを考えると比較的良好な局所制御率が得られたが、16例（46%）と高率に遠隔転移が出現し、5年全生存率は43%と満足できるものではなかった。遠隔転移16症例中、初回再発部位は傍大動脈リンパ節が10例（29%）と最も多くを占めていた。そのうち6例は治療後6か月以内に傍大動脈リンパ節転移が出現しており、潜在的な傍大動脈リンパ節転移陽性例であったと考えられた。局所制御率が向上するにしたがって、長期予後の改善のためには遠隔転移、特に傍大動脈リンパ節転移の対策が重要になってきたと考えられる。

- 1. 傍大動脈リンパ節に対する予防照射の合理的根拠

FIGO臨床病期のIII期における傍大動脈リンパ節転移の頻度は、リンパ節摘除術や生検による検討から約25-30%と報告されており、進行子宮頸癌の予後に影響を与える要因の一つである（文献6）。現在、局所進行子宮頸癌の標準治療となっている化学放射線治療の根拠となったアメリカでの第III相臨床試験では、傍大動脈リンパ節転移の有無は生検にて診断され、リンパ節転移症例は臨床試験から除外されている（文献1-3）。また傍大動脈リンパ節転移に関する診断をCTによる画像診断のみとしたカナダの第III相試験では、化学放射線治療による生存率の明らかな向上は得られていない。したがって傍大動脈リンパ節転移に関する診断がCTのみで行われた場合、潜在的なリンパ節転移の可能性がある症例に対して化学療法の併用により遠隔転移が抑制されるか、さらには生存率が向上するかについては十分に検証されておらず、最適な治療法に関してはまだ論議のあるところである。

傍大動脈リンパ節転移が顕在化した後は、多くの症例で他の遠隔転移が出現し、その予後は不良である。しかしながら初回治療時に傍大動脈リンパ節転移を有する症例でも同部位への放射線治療で治癒する例は約10-30%あり、また傍大動脈リンパ節への再発後も、症例を選べば放射線治療にて長期生存が可能となる症例がある（文献5,7,8）。以上から傍大動脈リンパ節転移例の必ずしも全てが全身転移となるわけではなく、局所治療である傍大動脈リンパ節領域への放射線治療が奏効する例があることが示唆される。

傍大動脈リンパ節転移の危険性が高い症例に対して、同部位への予防照射が予後を改善するかどうかに関して、これまでにアメリカ（RTOG 79-20）とヨーロッパ（EORTC）で以下の２つの大規模な第III相試験が行われている（文献12, 13）。その結果、全体的に見るとRTOG 79-20では予防照射により生存率の改善が得られたのに対して、EORTCの研究では予防照射の生存率への寄与は認められなかった。2つの研究を比較すると、RTOGに比してEORTCの方が進行症例の比率が高く、局所再発の頻度が明らかに高かった。このため局所が制御された症例に限ってみると、両者とも予防照射により遠隔転移の頻度は低下していた。ただし予防照射を行うと消化管の有害事象の発生頻度は明らかに増加し、特に腹部手術の既往がある場合、重篤な有害事象の発生頻度は10%程度と高かった。したがって局所進行子宮頸癌において傍大動脈リンパ節への予防照射は同部位への転移ひいては他の遠隔転移を低下させ、生存率の向上に寄与しうる可能性が示唆されたが、局所制御が十分に良好であること、および骨盤と傍大動脈リンパ節を合わせた広い領域への安全な照射法が確立されることが重要な条件と考えられた。

傍大動脈リンパ節領域に対する照射方法に関しては、IMRTを用いて正常組織の有害事象を軽減する試みが一部でなされているが、臨床応用に関する報告はまだほとんどない（文献14）。一方、これまでに行われた傍大動脈リンパ節領域に対する炭素イオン線治療としては局所進行膵癌に対する治療（プロトコール0204）がある。本プロトコールでは、膵臓の腫瘍から傍大動脈リンパ節領域までの範囲に総線量38.4GyE/12Fr～48.0 GyE/12Frを3週間で照射しているが、これまでに治療された28例中、消化管の早期反応はGrade 1が9例、Grade 2が5例にみられたのみで、Grade 3以上は出現していない。また消化管の遅発性反応に関してもGrade 1が6例、Grade 2が4例認められたが、Grade 3以上の重篤な有害反応は出現していない。これまでの子宮頸癌の臨床試験（9702、9902）の結果からはリンパ節に対する予防照射に要する線量は40 GyE程度で十分と考えられるので、傍大動脈リンパ節への40 GyE程度の予防照射は安全に施行できると考えられる。しかし骨盤と傍大動脈リンパ節を合わせた広い領域への炭素イオン線治療はこれまで行われたことがなく、その安全性は確認されていない。したがって本治療の安全性と効果は臨床試験で確認される必要がある。

以上をまとめると、１）局所進行子宮頸部扁平上皮癌に対する炭素イオン線治療で、化学放射線治療と同等以上の良好な局所制御が得られるようになった。２）しかし傍大動脈リンパ節転移を中心とした遠隔転移が高頻度に発生し、長期予後の改善は得られていない。これは潜在的に傍大動脈リンパ節転移の可能性が高い症例を治療の対象としているためと考えられる。３）このような症例に対する傍大動脈リンパ節領域への予防照射は、同部への再発を減少させるだけでなく、長期予後の改善に寄与しうる可能性がある。４）炭素イオン線治療は、Ｘ線による一般の放射線治療に比して正常組織への照射線量を低減することができるため、骨盤と傍大動脈リンパ節を合わせた領域へも比較的安全に照射することが可能と考えられる。したがって、これまで行ってきた骨盤部への炭素イオン線治療（プロトコール9902）に加えて、傍大動脈リンパ節領域に対する炭素イオン線による予防照射を行うことは、局所進行子宮頸部扁平上皮癌の長期予後の改善に寄与しうる可能性がある。本治療法の有効性を最終的に比較試験で検証することを目指して、まずその安全性と短期効果について第I/II相臨床試験で評価することは意義あることと考えられるため、本プロトコール治療を計画した。

４　目的と評価指標（エンドポイント）

- 1. 試験の目的

　局所進行子宮頸癌症例に対して炭素イオン線を用いて骨盤および傍大動脈リンパ節領域を同時に照射（１回3.0GyE、週４回法で39.0Gy）および、子宮の病巣を中心とした局所照射（2段階で縮小）を行った時の安全性と有効性を確認する．

- 1. 評価指標（エンドポイント）

4-2-1 主要評価指標（プライマリーエンドポイント）

1) 正常組織の早期反応（照射開始後90日以内）

　早期反応の評価には、「RTOG Acute Radiation Morbidity Scoring System」もしくは「NCI/CTC Common Terminology Criteria for Adverse Events, Version 3.0」を用いることを原則とする。

4-2-2副次的評価指標（セカンダリーエンドポイント）

1) 正常組織の遅発性反応（照射開始後91日以降）

　　 正常組織の遅発性反応の評価には、原則｢RTOG/EORTC Late Radiation Morbidity Scoring System｣を用いる。

　 2) 局所制御期間

　　　治療開始日から起算して治療体積内局所に腫瘍の再発あるいは再燃と判断された日までの期間を求め、Kaplan-Meier法により局所制御率として表示する。

　　3) 無再発生存期間

治療開始日より起算して再発と判断された日またはあらゆる原因による死亡日のうち早い方までの期間を求め、Kaplan-Meier法により無再発生存率として表示する。

５　対象の選択

本研究の対象患者は、以下の条件を満たさなければならない。

5-1適格条件

1. 生検（組織診）で証明された子宮頸部扁平上皮癌。

2. FIGOの臨床病期のIIB期で最大腫瘍径が4cm以上のもの、ないしIIIB期、IVA期。

3. 腹部CTにて腹部傍大動脈領域に短径1cm以上のリンパ節が描出されない。

4. 計測可能な病変である。

5. 年齢は80歳以下である。

6. PSは0～2である。

7. 十分な骨髄機能を有する。

　　白血球 > 3000/mm^3^

ヘモグロビン > 10g/dl

血小板 > 100,000/mm^3^

8. 6ヶ月以上の生存が見込まれる。

9. 文書による同意が得られている。

5-2 不適格条件

1. 重篤な合併症（例えば制御困難な心疾患・高血圧・糖尿病、難治性の感染症、急性期の消化性潰瘍、制御困難な精神病など）を有する。

2. 活動性の重複癌を有する。

3. 当該照射部位に放射線治療の既往がある。

4. 化学療法の既往がある。

5. 医学的、心理学的または他の要因により担当医師が不適当と考える。

６　インフォームド・コンセントと倫理審査

6-1インフォームド・コンセント

6-1-1説明

担当医師は試験実施にあたって、説明文書を用いて下記の内容について口頭で説明する。

- 炭素イオン線治療の副作用、効果をみるための臨床試験であること
- 本臨床試験の根拠、意義、必要性、目的など
- 本試験治療の方法と試験計画構成
- 期待される効果（利益）
- 予想される副作用（有害反応、危険性、治療関連死を含む）
- 替わり得る治療方法の有無、治療内容と予想される利益、危険性
- 試験に関わる費用
- 試験への参加は患者の自由であり、同意後もいつでもこれを撤回できること。試験への参加を断った場合または同意を撤回した場合でも、患者に治療上の不利益は生じないこと。
- 研究の成果が発表される場合、臨床試験の評価を実施する場合にも患者の人権、プライバシーは保護されること
- 質問の自由
- 試験への参加を中止させる場合の条件または理由
- 重粒子治療において患者の試験継続の意思に影響を及ぼすような新たに得られた情報の開示
- 患者がまもるべき事項（治療後の診察など）

　担当医師は、患者に説明した説明文書を渡し、患者が質問する機会と重粒子治療に参加するか否かの判断をするのに十分な時間を与えなければならない。また、質問に対して患者が満足するようにこたえなければならない。

　6-2同意の取得

　患者が臨床試行の内容をよく理解したことを確認した上で、本試験への参加について本人の意志を確認する。患者本人（未成年者では親権者も含む）が試験への参加に同意した場合は、説明・同意文書末尾の同意書に署名捺印を得る。同意書原本は放射線医学総合研究所内研究事務局に保管し、そのコピーを患者本人に手渡すとともにに患者病歴にも保存しておく。

6-3倫理審査および承認

　炭素イオン線治療開始前に適格性確認表、患者病歴、説明同意文書を臨床医学研究倫理審査放射線　治療部会に提出し、審査承認を受ける。

７　登録と追跡

7-1登録

　適格条件を満たし、かつ不適格条項に該当しない患者は、臨床医学研究倫理審査放射線治療部会で承認され、本プロトコール研究の対象患者として登録される。この際、以下の記録文書が、放医研に保管される。

１） 患者の同意・承諾書

２） 患者の適格性を示す文書（適格性確認表）

３） 患者の主訴、既往歴、現病歴などを示す文書（患者病歴）

４） 研究登録書

５） 臨床医学研究倫理審査放射線治療部会審査判定書

６） 臨床医学研究倫理審査放射線治療部会審査判定通知書

7-2追跡調査および調査期間

　登録された症例は全例が追跡対象となる。そのため、試験期間中は、患者が追跡調査を拒否した場合をのぞき、全例にたいして追跡調査を可能な限り行う。生死の確認、再発の有無、正常組織の遅発性反応の追跡調査期間は最終症例登録より２年間とする。予定追跡期間終了時点で、臨床研究班においてさらに追跡の延長が必要と判断された場合には、延長理由と期限を明記し、評価部会に承認を求める。担当医師および臨床研究班班員は、協力して「炭素イオン線治療の評価指針」（平成13年２月）に従い患者の診察および追跡調査を行なう。診察は、原則としてはじめの12カ月は月１回、その後は３カ月に１回、３年目以降は、半年に１回以上とする。

８　試験期間と予定症例数

8-1予定症例数

20例を目標とする。

8-2試験期間

　　登録期間は2006年4月１日より2008年3月31日までの2年間、追跡期間は登録終了後2年間とする。

8-3試験期間の延長

解析に足る十分な症例の登録が達成されなかった場合には、臨床研究班において期間延長につき検討する。

９　炭素イオン線治療

9-1使用機器

　炭素イオン線治療は放射線医学総合研究所重粒子医科学センターに設置された医用重粒子加速器（HIMAC)および照射装置を用いる。

9-2標的体積

9-2-1肉眼的腫瘍体積（GTV; gross tumor volume)

　GTVは、触診、視診、画像診断等により明らかに腫瘍が存在すると判断される領域の体積であり、触診、視診に加えてCT、MRI、PETなどの画像所見、膀胱鏡・直腸鏡所見を参考に設定される。

9-2-2臨床的標的体積（CTV：clinical target volume）

CTVは、上記のGTVを含む範囲に加えて潜在的な腫瘍の存在が考えられる領域であり、子宮全体、膣（少なくとも上部）、子宮傍結合織、ならびに骨盤リンパ節、腹部傍大動脈リンパ節が含まれる。骨盤リンパ節への照射では左右の内・外・総腸骨節および仙骨前節を十分に含める。腹部傍大動脈リンパ節への照射範囲は、大動脈・下大静脈およびその周囲組織を含み、腹側は血管の前方5 mm以内、背側は両側腸腰筋および椎体の前縁、上端はほぼL1の高さとする。

9-2-3計画標的体積（PTV：planning target volume）

PTVは、CTVに起こりうるすべての幾何学的な変動に不正確性を考慮した領域で、これらの誤差に対する安全域（セーフティマージン）は重要臓器に隣接する場合を除き原則として5mm以上とする。

9-3治療計画

9-3-１治療計画データ収集

治療計画はCTシミュレータにより連続撮影された3あるいは5 mmスライス厚CT画像を用いて３次元放射線治療計画を行う。CT撮影は呼吸同期装置を用いて呼気位相で行う。ただし呼吸性移動が無視できる場合はこの限りではない。

9-3-2治療体位および患者固定

　治療体位は、最も良好な線量分布と再現性が得られる体位が症例ごとに選択される。また垂直・水平固定ポートにおいても任意の角度での照射を可能にするため、原則として回転可能な患者固定用ベッドを用いる。照射位置の正確な再現のために熱可塑性樹脂等により作成された患者固定具を用いて照射領域の固定を行なう。

9-3-3線量計算

　連続撮影された CT 画像を使用して３次元治療計画を行なう。原則として治療計画の結果の客観的評価のために標的体積および重要臓器等のDVH（dose volume histogram）を算出する。

9-4炭素イオン線と照射野の整形

　炭素イオン線の整形は、照射軸方向には治療計画の情報を基にして作成されたボーラスにより行なう。側方は、主に照射系に付属の多葉コリメーターにより行なうが、照射野が小さくかつエネルギーが290MeV/μの場合には個別の患者コリメーターを製作して用いる。

9-５位置決め法

　照射位置の確認は、治療室位置決めコンピューターを用いて、照射軸方向とそれに直交して設置されたＸ線透視（撮影）装置のＸ線透視画像により行われる。このＸ線画像との比較のために、治療計画に使用されたCT画像による各治療方向およびそれと直交する再構成画像（DRR: digitally reconstructed radiograph）が用いられる。初回位置決め時に決定されたＸ線透視画像は位置決め基準画像として毎回の位置決めに用いられる。

9-６線量指示、線量分割、照射法

　炭素イオン線の拡大ブラーグピーク(SOBP: spread-out Bragg peak)はヒト由来培養腫瘍細胞系（HSG: human salivary gland腫瘍細胞）の生残率がSOBP内で一定になるように作られている。光子線に対する生物効果の相対比であるRBE（relative biological effectiveness）は、SOBP内の線量平均LETが80 keV/μmになる点（SOBPの遠位点から8 mm近位の点）で3.0となるように基準化されている。物理線量にRBEをかけた線量を光子等価線量（photon equivalent dose）と定義し、GyEの単位を用いる。SOBP内の光子等価線量は一定ではあるが、物理線量は一定ではないため、治療線量の物理線量はSOBPの中央での線量をあらわす。

骨盤部への治療は、まず子宮頸部の病巣から骨盤リンパ節まで含む領域に対して、1日1回3.0 GyE、2週間で6-8回を原則として、合計39.0 GyEの照射を行う（骨盤照射）。骨盤照射の上縁は総腸骨動静脈分岐部付近の高さとする。次いで照射範囲を子宮頸部の病巣から腫瘍浸潤の可能性が高い子宮傍結合織・子宮体部・膣上部、隣接リンパ節などに絞って、1日1回3.0 GyEで5回、合計15.0 GyEの照射を行う。最後に治療範囲を肉眼的腫瘍体積に絞り、消化管を完全に照射範囲から外して、1日1回9.0 GyEで2回、合計18.0 GyEの照射を行う。全分割回数は20回、全治療期間は5週間で、肉眼的腫瘍体積に対する総線量は72.0 GyEとなる。なお第2，第3段階ともCTを撮り治療計画を行うこととする。

傍大動脈リンパ節に対する治療は骨盤照射と同日に行う。傍大動脈リンパ節の治療範囲の上縁は原則としてL1の高さとし、下縁は骨盤照射の治療範囲の上縁と接するようにして、本領域に1日1回3.0 GyE、2週間で6-8回を原則として、合計39.0 GyEの照射を行う。

骨盤照射と傍大動脈リンパ節領域の照射は、前方・左方・右方からの3門で行い、前者の照射に引き続いて後者の照射も同一日に同一方向から行う。前方からの照射野のつなぎ目はほぼL5上縁の高さ、左右からの照射野のつなぎ目はほぼL5/S1の高さとし、両者の間隔は原則として2 cmとする。つなぎ目の部分の線量は指示線量の+ 5%を超えない範囲とする。骨盤照射と傍大動脈リンパ節領域の照射は、原則として呼吸同期照射を行うが、呼吸性移動が無視できる標的に対してはその限りではない。

9-7　治療内容

　治療研究は、安全性を確認（第I相）した後に、有効性の確認（第II相）に進む。6名に対して本治療を行い、安全性を確認する。当6名の中からNCI-CTCAE version 3の基準によるGrade III以上の非血液毒性ないしGrade IVの血液毒性が出現した場合は、プロトコール運営委員会を開き、傍大動脈リンパ節領域の線量の低減について検討する。安全性が確認された後（最初の3例が少なくとも6ヶ月以上経過し、第6症例終了後3ヶ月以上経過）に、有効性の確認に進む。

　骨盤内が制御され、かつ傍大動脈リンパ節領域内再発が生じた症例が2名（傍大動脈リンパ節制御率90%以下）以上出現した場合、プロトコール運営委員会を開きプロトコールの継続について検討する。

9-8　線量の制限

　これまでの9403, 9702の臨床試験にて得られた結果から、以下の臓器については、腫瘍の浸潤がある場合をのぞき、原則として以下に示す線量を超えない照射法をとる。

- 直腸・S状結腸 60 GyE/5週
- 小腸　　　50 GyE/5週
- 膀胱　　　全容積の1/3以上：40 GyE/4週、膀胱後方1/3以下：70 GyE/5週
- 腎臓　　　全容積の1/2以上：10 GyE/3週、全容積の1/2以下：30 GyE/3週

9-9　治療中、治療後の他の治療の併用について

　炭素イオン線治療中および治療後でも再発・転移がない場合には化学療法の併用は行わない。ただし治療に伴う有害反応に対する治療は制限しない。

10　病理

10-1初回の病理標本

　生検標本(HE）染色、可能であればブロック）は、紹介医の所属する施設または放医研の病理医により診断される。なお、切除標本の場合もこれに準ずる。

10-2治療後の病理標本

　炭素イオン線治療後にプロトコールの対象腫瘍に対して生検または外科的切除が行われた場合は、その病理標本は当該施設の病理医により検査される。放医研以外の施設で行われた場合は、代表的なスライド標本および病理レポートのコピーは放医研に提供され、検討されることが望ましい。

11　必要な評価項目および検査

11-1治療開始前検査

　治療前評価は紹介医の所属する施設または放医研において行なわれる。治療医は、紹介医により紹介された患者について、報告所見を確認し必要な検査を要請するか、または放医研においてこれを追加する。治療前の評価項目は以下のとおりである。ただし、造影剤等の薬剤にアレルギーを有する患者など臨床上問題となる場合には、必ずしもすべての検査を行なう必要はない。

１） 病歴

２） 自覚症状

３） 身体所見

４） 臨床検査

a）血液一般・血液像

b）血清生化学的検査

c）検尿

　d）腫瘍マーカー（SCC, CEAなど）

５） 画像診断：単純Ｘ線・CT・ MRI・PETなど

６） 膀胱鏡検査、直腸鏡検査など、病変の進展範囲決定に必要な諸検査

11-2治療期間中および観察期間、追跡期間中の評価・検査

　炭素イオン線治療開始日から90日までの間を観察期間とする。観察期間終了から登録した症例が全例、追跡終了となる日までを追跡期間とする。

11-2-1治療期間中および観察期間、追跡期間中の検査

　治療期間中は原則として２週に１回以上、観察期間中は２ヶ月に１回以上、追跡期間中は６ヶ月に１回以上、血液一般・血液像、血清生化学的検査を行う。

11-2-2治療期間中および観察期間、追跡期間中の臨床所見のチェック

　治療期間中は原則として２週に１回以上、観察期間中は1ヶ月に１回以上、追跡期間中は６ヶ月に１回以上、臨床所見のチェックを行う。観察期間中は｢RTOG Acute Radiation Morbidity Scoring System｣もしくは「NCI Common Terminology Criteria for Adverse Events, Version 3.0」、追跡期間中については｢RTOG/EORTC Late Radiation Morbidity Scoring System｣に従いスコアリングを行い、最も強い反応gradeをそれぞれ急性および遅発性反応とする。

11-3腫瘍縮小効果判定のための検査

　治療終了後、観察期間中に１度、腫瘍縮小効果判定のためのCTあるいはMRIによる画像診断を行う。観察期間終了後は追跡期間終了まで６ヶ月に１度程度CTあるいはMRIによる画像診断を行うことを原則とする。

12　予期される有害反応と対策、治療変更基準

12-1予期される有害反応

｢RTOG Acute Radiation Morbidity Scoring System｣、「NCI Common Terminology Criteria for Adverse Events, Version 3.0」、｢RTOG/EORTC Late Radiation Morbidity Scoring System｣に示される急性あるいは遅発性反応が発生する可能性がある。

12-2炭素イオン線治療の変更・中止基準

　炭素イオン線治療中に｢RTOG Acute Radiation Morbidity Scoring System｣ないし「NCI Common Terminology Criteria for Adverse Events, Version 3.0」におけるGrade 3以上の非血液毒性ないしGrade 4の血液毒性が観察された場合には、プロトコール運用委員会にはかり投与線量ならびに試験の継続について検討する。また上記の血液・非血液毒性が観察された場合には、担当医の判断により治療の続行につき検討し、適切な処置を行う。その他、機械の故障、患者の拒否、あるいは腫瘍の進行、患者状態の変化などにより担当医が必要と認めた場合には、炭素イオン線治療を休止するか、または他の放射線を用いた治療や放射線以外の治療に振り替える。患者の申し出による中止変更を除いて担当医師は、患者にその理由を説明する。またプロトコール運用委員会に変更内容あるいは中止の経緯について詳細に報告する。

12-3有害反応／有害事象に対するその他の対処方法

　｢RTOG Acute Radiation Morbidity Scoring System｣、「NCI Common Terminology Criteria for Adverse Events, Version 3.0」、｢RTOG/EORTC Late Radiation Morbidity Scoring System｣に示される急性あるいは遅発性反応が発生した可能性がある場合には、慎重に症状の推移を観察記録すると同時に適切な検査処置治療を行う。

12-４　予期できない有害反応／有害事象に対する対処方法

　これまでの炭素イオン線治療において報告されていない重篤な有害反応／有害事象が発生し、治療との因果関係が否定できない場合には、あるいは予期される有害反応の発生頻度が予測よりも高い場合には、すみやかにプロトコール運用委員会で検討し、必要に応じて研究班会議を召集し、詳細な内容の報告を行う。必要に応じてネットワーク会議にはかり、同意説明文書の改定や患者への説明を行う。

13 炭素イオン線治療終了後の治療

　治療終了後は、対象腫瘍の再燃、再発が証明されるまでは重粒子線治療部位に対する一切の追加治療は行なわない。対象腫瘍の再燃、再発時には、担当医師の判断において追加治療を行なうことができる。その際の治療法に制限はない。腫瘍再燃、再発の診断は可能な限り生検により行われるべきであるが、生検による証明が不可能な場合は、画像診断および臨床所見により確定診断を行ってよい。ただし、これらの場合、治療内容等は患者追跡調査記録用紙に詳細に記載されるものとする。他臓器の腫瘍、遠隔転移に対する治療は、プロトコールの対象腫瘍に対する影響の如何を問わず行なうことができる。

14　定期的な臨床試験の評価と報告

14-1目的、時期、結果の報告

　試験が安全に行われているか、データが正確に収集されているか、試験がプロトコールの規定通りに実施されているか、効果が期待通りかどうかを確認する目的で、原則として年２回臨床研究班会議を行う。

14-2臨床試験の評価項目

- 症例集積達成状況；登録症例数、累積／期間別
- 適格／不適格例
- 病理診断
- 炭素イオン線治療終了状況
- 登録時患者背景因子
- 急性反応・遅発性反応（有害反応）発生状況
- 再燃・再発／転移の発生状況

15　登録の終了と中止およびプロトコール改訂

15-1症例登録の終了

登録症例が予定症例数に達した事を臨床研究班会議で確認された時点で登録を終了する。

15-2試験の中止

｢RTOG Acute Radiation Morbidity Scoring System｣、「NCI/CTC Common Terminology Criteria for Adverse Events, Version 3.0」、｢RTOG/EORTC Late Radiation Morbidity Scoring System｣のスコアにおいてGrade 3 以上の正常組織反応が2例以上（2/20、10%）で観察された場合、あるいはそれ以外のなんらかの理由により、本プロトコール治療の続行に疑念が生じた場合には、プロトコール運用委員会で討議の上、必要に応じて臨床研究班を開催し、本プロトコール治療の適用の中断について討議し、中断の判断となった場合はその根拠、理由を重粒子線治療ネットワーク会議評価部会に報告する。評価部会ではそれを検討して、本プロトコール治療の中止の是非を決定し、ネットワーク会議の承認を得る。担当医師は、患者に対し、治療の続行に疑念の生じた旨説明を行う。

16　報告義務のある有害反応／有害事象

16-1　有害反応／有害事象の報告義務

「重篤な有害反応」と「予期されない有害事象」が生じた場合、担当医は報告用紙（別紙）に記載の上研究事務局／プロトコール運用委員会に報告する。

- 1. 担当医の報告義務

　16-2-1急送報告

- 一次報告：担当医は急送報告の対象となる有害事象がおきた場合、有害事象を知りえてより72時間以内に「炭素イオン線AE/AR急送一次報告書」に所定事項を記入し、研究事務局／プロトコール運用委員会に提出する。
- 二次報告：さらに「炭素イオン線AE/AR報告書」に所定事項を記入し、より詳しい情報を記入した「症例報告の詳細（A4自由形式）」を別紙として作成し、両者を７日以内に提出する。その際、提出期限が優先するのでまだ記入できない段階の情報は空白でよい。
- 三次報告：原則として「炭素イオン線AE/AR報告書」の所定の事項をすべて記入し、有害事象を知りえてより15日以内に研究事務局／プロトコール運用委員会にコピーを提出する。「症例報告の詳細」に追加する項目があれば追加して併せて送付する。
- 追加報告：死亡の場合の剖検報告書など、三次報告以降に得られた情報や三次報告でまだ未記入

　　があった場合の追加などの追加報告がある場合、それぞれ適切と思われる書式を用いて研究事務局／プロトコール運用委員会に報告する。

16-2-2通常報告

- 予期されないGrade 3の有害事象：「12-1予想される有害反応」に記載されないすべてのGrade 3の有害事象が、急送報告に準じた「通常報告」の対象となる。

17　解析関連事項

17-1評価指標（エンドポイント）の定義と解析方法

17-1-1局所制御の定義

　照射野内（計画標的体積内）に明らかな腫瘍の再発、再燃、あるいは新たな病変の出現を認めないことを局所制御と定義し、直接法、又は、Kaplan-Meier法によって算出した局所制御率（対象病変が局所制御されている割合）をもって表示する。局所再発は、治療により消失した腫瘍がふたたび出現した場合あるいは残存していた腫瘍が、画像診断上（CTあるいはMRI等）明らかな腫瘍サイズの増大を認めたものとする。可能であれば、生検により組織学的に確認されることが望ましい。

17-1-2生存期間

　　　　治療開始日から起算して治療体積内局所に腫瘍の再発あるいは再燃と判断された日までの期間を局所制御期間とし、Kaplan-Meier法により局所制御率として表示する。また治療開始日より起算して再発と判断された日またはあらゆる原因による死亡日のうち早い方までの期間を無再発生存期間とし、Kaplan-Meier法により無再発生存率として表示する。

17-2解析時の症例の取り扱い

「日本癌治療学会・癌規約総論（第１版）」に基づいて、以下のように定める。

注１.不適格例（除外例）：適格症例として登録後に、不適格であることが判明した場合には、臨床研究班で検討した上、重粒子線治療ネットワーク会議評価部会において決定し、その症例を除外例とし解析の対象からはずす。

注２.不完全治療例：中止例、脱落例、観測不備例がある。

　　中　止　例： 安全性に関連して医師または患者の判断によって治療を中止した症例

医師側の判断による治療の打ち切り

患者側の判断による治療の打ち切り

　　脱　落　例： 特に安全性とは無関係に、計画された治療が行われなかった症例

医師側の治療逸脱

　　　　　　　 患者側の都合による打ち切り

　　　　　　　 患者側の治療不遵守

　　　観測不備例：何らかの理由により治療開始後、効果を確認するために必要な病変の測定が行われなかった症例

　　　　　　　　観察測定不可能

　　　　　　　　観察測定欠落

注3.解析除外例：個々の不完全治療例において、解析対象とするかどうかについては、臨床研究班において検討し、重粒子線治療ネットワーク会議評価部会において決定する。但し、以下の場合は、原則として抗腫瘍効果の解析対象からは除外する。

　　(1) 照射の休止が連続して２週間（１４日）を越えた場合。

　　(2) 総治療線量が当初計画の９０%に満たない場合。

　　(3) 他の放射線による治療に振り替え、それによる治療線量が総治療線量の１０%を越えた場合。

　　(4) 放射線以外の治療法に切り替えた場合。

注4.中途打ち切り例(Censoring case)：解析対象例の内、解析施行時点で死亡が確認されていない症例をいう。この中には、研究の時間切れによる中止例(Withdrawal)と消息不明例(Lost to follow-up)があり、それぞれの数および理由を明記しておく。

注5.不適格例、不完全治療例、解析除外例等については、その数、内容等についても明記する。

　　注6.評価項目毎に解析対象例の登録症例数に対する割合についても明記する（８５%以上であることが好ましい）。

　　　　　　　 登録例

適格例　　　　　不適格例　　　　　研究班　　　除外例

完全治療例　　　不完全治療例　　　 研究班　　 解析除外例

　　　　　　　解析対象例

　　　 死亡例　　　　　　　　　 中途打ち切り例

　　　　　　　　　　 時間切れ生存例　　　　　 消息不明例 　　　 その他の理由

18　研究組織

　本研究は、文部科学省重粒子線治療プロジェクト研究、重粒子線治療婦人科臨床研究班「婦人科腫瘍に関する炭素イオン線治療研究」で、文部科学省重粒子線治療プロジェクト研究の研究費によって運営される。本臨床試験計画書は、倫理面については放射線医学総合研究所臨床医学研究倫理審査委員会の審査を受けたものであり、重粒子線治療ネットワーク会議の承認を得たものである。試験は、本臨床試験計画書を遵守して行われるが、その実施中は、重粒子線治療ネットワーク会議評価部会の定期的なモニタリングを受けその指示、勧告に従うものとする。

　18-1研究代表者

鈴木通也

放射線医学総合研究所　特別研究員

TEL : (043)206-3360　　FAX: (043)256-6506

18-2研究事務局

加藤真吾

放射線医学総合研究所・重粒子医科学センター

〒263　千葉市稲毛区穴川4-9-1

TEL : (043)206-3350　　FAX: (043)256-6506

19　参考文献

1) Eifel PJ, Winter K, Morris M, et al. Pelvic irradiation with concurrent chemotherapy versus pelvic and para-aortic irradiation for high-risk cervical cancer: An update of Radiation Therapy Oncology Group Trial (RTOG) 90-01. JCO 2004; 22: 872-880.

2) Rose PG, Bundy BN, Watkins EB, et al. Concurrent cisplatin-based radiotherapy and chemotherapy for locally advanced cervical cancer. New Engl J Med 1999; 340: 1144-1151.

3) Whitney CW, Sause W, Bundy BN, et al. Randomized comparison of fluorouracil plus cisplatin versus hydroxyurea as an adjuvant to radiation therapy in stage IIB-IVA carcinoma of the cervix with negative para-aortic lymph nodes: A Gynecologic Oncology Group and Southwest Oncology Group Study. JCO 1999; 17: 1339-1348.

4) Green JA, Kirwan JM, Tierney JF, et al. Survival and recurrence after concomitant chemotherapy and radiotherapy for cancer of the uterine cervix: A systematic review and meta-analysis. Lancet 2001; 358: 781-786.

5) Nakano T, Kato S, Ohno T, et al. Long-term results of high-dose rate intracavitary brachytherapy for squamous cell carcinoma of the uterine cervix. Cancer 2005; 103: 92-101.

6) Lagasse LD, et al. Results and complications of operative staging in cervical cancer. Experience of the Gynecologic Oncology Group. Gynecol Oncol 1980; 9: 90-98.

1. Varia MA, Bundy BN, Deppe G, et al. Cervical carcinoma metastatic to para-aortic nodes: Extended field radiation therapy with concomitant 5-fluorouracil and cisplatin chemotherapy: A Gynecologic Oncology Group study. Int J Radiat Oncol Biol Phys 1998; 42: 1015-1023.
2. Niibe Y, Nakano T, Ohno T, et al. Prognostic significance of c-erbB-2/HER2 expression in advanced uterine cervical carcinoma with para-aortic lymph node metastasis treated with radiation therapy. Int J Gynecol Cancer 2003; 13: 849-855.
3. Pearcey R, Brundage M, Drouin P, et al. Phase III trial comparing radiotherapy with and without cisplatin chemotherapy in patients with advanced squamous cell carcinoma of the cervix. JCO 2002; 20: 966-972

10) Rotman M, Pajak TF, Choi K, et al. Prophylactic extended-field irradiation of para-aortic lymph nodes in s stage IIB and bulky IB and IIA cervical carcinomas. Ten-year treatment results of RTOG 79-20. JAMA 1995; 274: 387-393.

11) Haie C, Pejovic MH, Gerbaulet A, et al. Is prophylactic para-aortic irradiation worthwhile in the treatment of advanced cervical carcinoma? Results of a controlled clinical trial of EORTC radiotherapy group. Radiother Oncol 1988; 11: 101-112.

12) Portelance L, Chao C, Grigsby PW, et al. Intensity-modulated radiation therapy (IMRT) reduces small bowel, rectum, and bladder doses in patients with cervical cancer receiving pelvic and para-aortic irradiation. Int J Radiat Oncol Biol Phys 2001; 51: 261-266.

資料１.患者の同意・承諾書 （同意説明文書）

資料２.患者の適格性を示す文書（適格性確認表）

資料３.患者の主訴、既往歴、現病歴などを示す文書（患者病歴）

資料４.研究登録書

資料５.臨床医学研究倫理審査放射線治療部会審査判定書

資料６.臨床医学研究倫理審査放射線治療部会審査判定通知書

資料７.重粒子線治療婦人科臨床研究班

資料８.重粒子線治療計画部会婦人科分科会

資料９.重粒子線治療ネットワーク会議評価部会

資料１０.重粒子線治療ネットワーク会議計画部会

資料１１.重粒子線治療ネットワーク会議

資料１２.放射線医学総合研究所臨床医学研究倫理審査放射線治療部会

資料１３.放射線医学総合研究所臨床医学研究倫理審査委員会

資料１４. RTOG Acute Radiation Morbidity Scoring System

資料１５.RTOG/EORTC Late Radiation Morbidity Scoring System

資料１６.炭素イオン線AE/AR急送一次報告書

資料１７.炭素イオン線AE/AR報告書

（資料５～１７：１１．別添資料参照）
