# Supplementary material for: Clinical Trial of Prophylactic Extended-Field Carbon-Ion Radiotherapy for Locally Advanced Uterine Cervical Cancer (Protocol 0508)
Source: PLoS One. 2015 May 20;10(5):e0127587. doi: 10.1371/journal.pone.0127587 (PMC4439043; doi:10.1371/journal.pone.0127587)
Supplement: S2 Protocol — (DOCX) [file pone.0127587.s003.docx]

**A phase I/II clinical trial of prophylactic extended-field radiotherapy of pelvic and para-aortic lymph nodes in locally advanced squamous cell carcinoma of the uterine cervix using carbon-ion beams（Cervix IV: Protocol 0508）**

Project leader: Michiya Suzuki

Project organizer: Shingo Kato

Research Center Hospital for Charged Particle Therapy, National Institute of Radiological Sciences

4-9-1 Anagawa, Inage-ku, Chiba-city, Chiba, 263-8555 JAPAN

Protocol

1st edition 2005/11/24

2nd edition 2006/1/7

3rd edition 2006/2/15

**Study Outline**

1. **Research brief**
   1. Objectives
   2. Study design
   3. Patient selection
   4. Carbon-ion radiotherapy (C-ion RT)
   5. Endpoints
2. **Study design**
   1. Study design schema
   2. Study design details
3. **Background**
   1. Current treatment for locally advanced uterine cervical cancer and C-ion RT
   2. Our previous clinical trials with C-ion RT
   3. Prophylactic radiation therapy for para-aortic lymph node regions
4. **Objectives and Endpoints**
   1. Study objectives
   2. Endpoints
5. **Protocol Eligibility Criteria**
   1. Patient eligibility criteria
   2. Patient ineligibility criteria
6. **Informed consent and IRB approval**
   1. Informed consent
   2. IRB approval
7. **Registration and follow-up**
   1. Registration
   2. Follow-up
8. **Sample size and patient accrual period**
   1. Sample size
   2. Patient accrual period
   3. Period extension
9. **Carbon-ion radiotherapy**
   1. Equipment
   2. Target volume
   3. Treatment planning
   4. Carbon-ion beam and field formation
   5. Patient positioning
   6. Prescribing dose, fractions and methods
   7. Study plan
   8. Dose constraints
   9. Other therapy treatments during or after C-ion RT
10. **Pathology**
    1. Tumor specimens before treatment
    2. Tissue or tumor specimens after C-ion RT
11. **Required evaluation**
    1. Pre-treatment evaluation
    2. Evaluation of observation and follow-up period
    3. Evaluation of tumor response and failure
12. **Expected adverse reaction, management of adverse reaction, and treatment modification**
    1. Expected adverse reaction
    2. C-ion RT modification
    3. Adverse reaction management
    4. Unexpected adverse reaction
13. **Additional treatments after C-ion RT**
14. **Clinical trial evaluation and report**
    1. The Working Group of the Gynecological Tumor
    2. Clinical trial assessment
15. **Trial registration expiration**
    1. Expiration
    2. Trial termination
16. **Reporting requirements of adverse event / adverse reaction**
    1. Reporting requirements of adverse event / adverse reaction
    2. Reporting requirements by attending physicians
17. **Statistical considerations**
    1. Definition of endpoint and method of analysis
    2. Handling cases for analysis
18. **Study team**
    1. Project leader
    2. Project organizer
19. **References**

1 Research brief

1-1 Objectives

To evaluate the efficacy and toxicity of prophylactic extended-field C-ion RT for locally advanced squamous cell carcinoma of the uterine cervix.

1-2 Study design

Phase I / II clinical trial

1-3 Patient selection

1-3-1 Patient eligibility criteria

1. Pathologically proven squamous cell carcinoma of the uterine cervix

2. International Federation of Gynecology and Obstetrics (FIGO 1994) Stage IIB with tumor size > 4 cm in diameter, III, or IVA disease without rectal invasion

3. Patient does not have para-aortic lymph nodes > 1 cm in minimum diameter on CT image

4. Tumor must be grossly measurable

5. Age < 80 years

6. World Health Organization performance status < 3

7. Adequate bone marrow function; WBC > 3000 cells/mm^3^, Platelets > 100,000 cells/mm^3^, Hemoglobin > 10.0 g/dl

8. Estimated life expectancy > 6 months

9. Patient provides written informed consent to participate in this study before initiation of therapy.

1-3-2 Patient ineligibility criteria

1. Severe co-morbidity; severe pelvic infection, severe psychological illness etc.

2. Active synchronous cancer

3. Prior radiotherapy to the pelvic or para-aortic region

4. Prior chemotherapy

5. Medical, psychological or other factor deciding ineligibility by attending physician

1-4 Carbon-ion radiotherapy (C-ion RT)

C-ion RT delivered by HIMAC at NIRS. The treatment consisted of extended-field irradiation of 39.0 gray equivalents (GyE) in 13 fractions, and additional 15.0 GyE in 5 fractions was given to the gross tumor volume (GTV) and surrounding tissues. With regard to local boost, 18.0 GyE in 2 fractions was given to GTV only. Total dose to the cervical tumor was 72.0 GyE over 20 fractions.


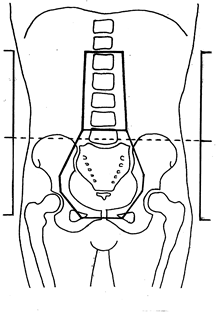


1-5 Endpoints

- - 1. Primary endpoint

1)Acute toxicity

1-5-2 Secondary endpoint

1) Late toxicity

2) Local control rate

3) Overall survival rate

4) Progression-free survival rate

1-6 Periods of patient accrual and follow-up, and sample size

1) Period of patient accrual: 2 years (April 2006 – May 2008)

Follow-up period: 2 years

2) Sample size: 20 cases

2 Study design

2-1 Study design schema

Figure 1

2-2 Study design details

- Attending physician confirms eligibility of each patient. If the patient has some problems, the patient must be re-confirmed by The Working Group of the Gynecological Tumor.
- All patients provide written informed consent to participate in this study before the initiation of therapy.
- After the patient provides written informed consent, the attending physician performs treatment planning. Dose prescription and dose distribution are confirmed by Treatment Planning Conference at NIRS.
- Attending physician submits eligibility check lists, patient summary, and written informed consent criteria to the Division for Radiation Therapy of IRB at NIRS and receives IRB approval before the start of treatment.
- Attending physician evaluates tumor response and local control after the end of treatment according to “Evaluation Guidelines of Carbon-ion Radiotherapy”.
- Attending physician evaluates acute and late toxicities after the end of treatment according to “Evaluation Guidelines of Carbon-ion Radiotherapy”.
- Attending physician will change the prescribed dose according to discussions of The Working Group of the Gynecological Tumor.
- If patient develops local or distant failure, attending physician will perform salvage treatment according to “13 Additional treatments after C-ion RT”.
- If patient develops acute or late complications, attending physician will treat according to “12 Expected adverse reaction, management of adverse reaction, and treatment modification”.
- Disease status and toxicities of each patient are periodically monitored and assessed after treatment.

Figure 1 Study design schema


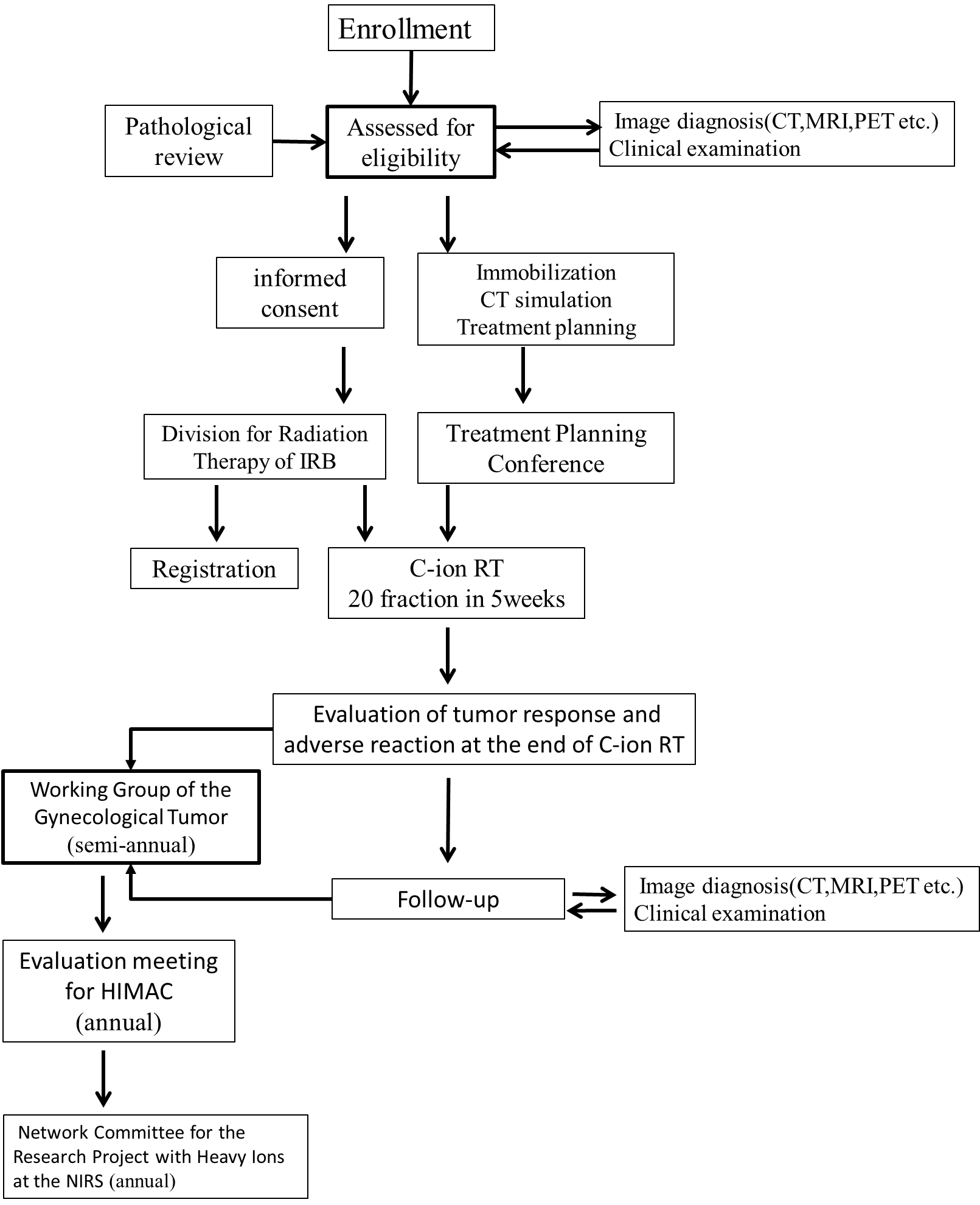


3. Background

3-1 Current treatment for locally advanced uterine cervical cancer and C-ion RT

Cisplatin-based concurrent chemoradiation therapy (CCRT) has become the standard treatment for locally advanced cervical cancer on the basis of several randomized phase III clinical trials in the 1990s ([1-4](#_ENREF_1)). Concurrent chemoradiation therapy improved survival benefit and local control for this disease. Nevertheless, a 5-year local failure rate of 30% or more has been observed, especially in patients with stage III or IVA disease, with the pelvis being the major site of failure ([1](#_ENREF_1)). Intracavitary brachytherapy plays an important role in the treatment of patients with uterine cervical carcinoma. However, intracavitary brachytherapy might not deliver a sufficient dose to extensive or bulky tumors, possibly resulting in local failure for patients with locally advanced disease ([5](#_ENREF_5)).

In 1994, clinical studies of C-ion RT were started at the National Institute of Radiological Sciences (NIRS). Carbon-ion beams possess improved dose localization properties, and this potentiality can produce great effects on tumors while minimizing normal tissue damage. Moreover, they possess a biological advantage due to their high relative biological effectiveness in the Bragg Peak. Several reports have demonstrated the favorable results of C-ion RT in the treatment of malignant tumors. Regarding C-ion RT for cervical cancer, four clinical trials were reported prior to the present study. Taken together, C-ion RT is expected to provide a higher local control rate for locally advanced bulky cervical cancer without concurrent chemotherapy because of the biological advantage of carbon-ion beams.

3-2 Our previous clinical trials

Since 1995, we have conducted four clinical trials for locally advanced squamous cell carcinoma or adenocarcinoma of the cervix (Protocol 9403, 9702, 9704 and 9902). The purpose of these trials was to evaluate the toxicity and efficacy of C-ion RT and to confirm its optimum dose for uterine cervical cancer, and the outcomes of these four clinical trials were reported. This review presents the results of these clinical trials. Protocol 9902 was a dose-escalation study of C-ion RT alone to the whole pelvis, and was designed for bulky tumors. The local control rates in patients with 64.0 – 68.8 GyE (23 cases) and 72.0 – 72.8 GyE (13 cases) were 61% and 92%, respectively All patients receiving 72.0 GyE, although not many in total number, showed local control, suggesting that C-ion RT alone has the potential to improve local control for locally advanced bulky cervical cancer with a total dose of 72.0 GyE. Despite the better local tumor control in previous trials, however, distant metastases frequently occurred, with 29% of patients in 2 trials (9702 and 9902) developing para-aortic lymph node (PALN) failure, and the overall survival rate was still unsatisfactory.

3-3 Prophylactic radiation therapy for para-aortic lymph node regions

PALN involvement occurs in a significant portion of patients with uterine cervical cancer, increasing in frequency with more advanced stage. The prevalence of PALN metastasis in locally advanced cervical cancer is 25-30% ([6](#_ENREF_6)). Several researchers have demonstrated the benefits of prophylactic extended-field irradiation (EFRT) for patients with high-risk cervical cancer ([5](#_ENREF_5),[7](#_ENREF_7),[8](#_ENREF_8)). However, pelvic radiation therapy with concurrent chemotherapy improved survival among patients with locally advanced cervical cancer compared with patients treated with EFRT without a chemotherapy arm, with the survival rate being significantly higher than that of the EFRT arms in RTOG 90-01 ([1](#_ENREF_1)). Since then, CCRT has become the standard treatment for locally advanced cervical cancer. Although CCRT had shown improvement of local control and overall survival against RT alone, around 10 - 15% of patients developed PALN metastasis after CCRT ([1](#_ENREF_1),[9](#_ENREF_9)). Therefore, several clinical trials conducted EFRT combined with concurrent chemotherapy, but those trials showed an unacceptably high rate of both acute and late severe toxicities ([10](#_ENREF_10),[11](#_ENREF_11)). In those trials, EFRT targeting PALN was delivered by conventional technique, and thus another strategy was needed for improved tumor control by prophylactic EFRT in patients with high-risk cervical cancer, such as intensity-modulated radiation therapy (IMRT) ([12](#_ENREF_12)) and C-ion RT. For this reason, we conducted another clinical trial of C-ion RT for locally advanced cervical cancer, this time using extended-field irradiation without concurrent chemotherapy (Protocol 0508).

4 Objectives and endpoints

4-1 Study objectives

To evaluate the efficacy and toxicity of prophylactic extended-field C-ion RT for locally advanced squamous cell carcinoma of the uterine cervix.

4-2 Endpoints

4-2-1 Primary endpoint

Acute toxicities: Acute toxicities will be evaluated according to “RTOG Acute Radiation Morbidity Scoring System” or “National Cancer Institute Common Terminology Criteria for Adverse Events, Version 3.0”.

4-2-2 Secondary endpoints

1) Late toxicities: Late toxicities will be evaluated according to “RTOG/EORTC Late Radiation Morbidity Scoring System”.

2) Local control rate, overall survival rate and progression-free survival rate: These will be evaluated by Kaplan-Meier method.

5 Protocol eligibility criteria

5-1 Patient eligibility criteria

1. Pathologically proven squamous cell carcinoma of the uterine cervix

2. International Federation of Gynecology and Obstetrics (FIGO 1994) Stage IIB with a tumor size > 4 cm in diameter, III, or IVA disease without rectal invasion

3. Patient does not have para-aortic lymph nodes > 1 cm in minimum diameter on CT image.

4. Tumor must be grossly measurable.

5. Age < 80 years

6. World Health Organization performance status < 3

7. Adequate bone marrow function; WBC > 3000 cells/mm^3^, Platelets > 100,000 cells/mm^3^, Hemoglobin > 10.0 g/dl

8. Estimated life expectancy > 6 months

9. Patient provides written informed consent to participate in this study before the initiation of therapy.

5-2 Patient ineligibility criteria

1. Severe co-morbidity; severe pelvic infection, severe psychological illness etc.

2. Active synchronous cancer

3. Prior radiotherapy to the pelvic or para-aortic region

4. Prior chemotherapy

5. Medical, psychological or other factor deciding ineligibility by attending physician

6. Informed consent and IRB approval

6-1 Informed consent

Written informed consent is mandatory to enter this study.

6-2 IRB approval

Attending physician submits eligibility check lists, patient summary, and written informed consent criteria to the Division for Radiation Therapy of IRB at NIRS and receives IRB approval before the start of treatment.

7 Registration and follow-up

7-1 Registration

When patients satisfy the Protocol Eligibility Criteria and are approved by the Division for Radiation Therapy of IRB at NIRS, the patients will be registered in this clinical trial. The following documents are stored at NIRS.

1. Written informed consent
2. Eligibility check lists
3. Patient summaries
4. Registration forms
5. Written findings by the Division for Radiation Therapy of IRB at NIRS
6. Notification of written findings by the Division for Radiation Therapy of IRB at NIRS

7-2 Follow-up

After completion of C-ion RT, patients were followed up every 1-3 months for 2 years, and every 3 or 6 months thereafter. Tumor response, local control, and toxicities will be evaluated according to “Evaluation Guidelines of Carbon-ion Radiotherapy”. Recurrences were defined as no evidence of tumor regrowth or recurrence in the treatment volume according to physical examination, CT, MRI, PET, and/or biopsy.

8 Sample size and patient accrual period

8-1 Sample size

20 cases

8-2 Patient accrual period

2 years (2006. 4.1.－2008.3.31.); Follow-up period was 2 years after registration.

8-3 Period extension

If registration is not sufficient for analysis, The Working Group of the Gynecological Tumor will discuss extending the period.

9 Carbon-ion radiotherapy

9-1 Equipment

C-ion RT is delivered by HIMAC at NIRS.

9-2 Target volume

9-2-1 Gross tumor volume (GTV)

GTV is defined by clinical examination, CT, MRI, PET, cystoscopy and rectoscopy.

9-2-2 Clinical target volume (CTV)

CTV includes all areas of gross and potentially microscopic disease, consisting of the tumor, uterus, ovaries, parametrium, at least the upper half of the vagina, para-aortic lymph nodes and pelvic lymph nodes (common iliac, internal iliac, external iliac, obturator and presacral lymph nodes).

9-2-3 Planning target volume (PTV)

PTV includes CTV plus a 5-mm safety margin for positioning uncertainty.

9-3 Treatment planning

9-3-1 CT simulation

Treatment planning is based on a set of 3- or 5-mm-thick CT images. CT imaging is performed by using a gating aspirator system.

9-3-2 Patient’s position and immobilization

Patients are positioned in customized cradles and immobilized with a low-temperature thermoplastic sheet.

9-3-3 Treatment planning

Three-dimensional treatment planning is performed on CT images, and is analyzed for dose-volume histogram for the target volume and organs at risks.

9-4 Carbon-ion beam and field formation

The carbon-ion beam is formed by bolus according to treatment planning. The carbon-ion field is formed by multi-leaf collimator or patient collimator.

9-5 Patient positioning

At every treatment session, the patient is positioned on the treatment couch with the immobilization devices, and the patient’s position is verified with a computer-aided, on-line positioning system.

9-6 Prescribing doses, fractions and methods

The radiation dose is calculated for the target volume and surrounding normal structures and is expressed in GyE, defined as the carbon physical dose (Gy) multiplied by a relative biologic effectiveness value of 3.0.

The treatment consists of prophylactic extended-field irradiation and local boost. Planning CT scan is basically performed three times during the course of the treatment, and CTV of local boost is reduced twice in accordance with tumor shrinkage. CTV of extended-field irradiation includes all areas of gross and potentially microscopic disease, consisting of the tumor, uterus, ovaries, parametrium, at least the upper half of the vagina, para-aortic lymph nodes and pelvic lymph nodes (common iliac, internal iliac, external iliac, obturator and presacral lymph nodes) (CTV-1), with a superior field border at the space between L1 and L2. PTV-1 includes CTV-1 plus a 5-mm safety margin for positioning uncertainty. CTV-1 is covered by at least 90% of the prescribed dose. After completing CTV-1 irradiation, first reduction of CTV includes GTV and uterine cervix, uterine corpus, parametrium, upper half of the vagina, ovaries and swelling lymph nodes (=CTV-2). A 5-mm margin is added to PTV-2. Finally, CTV is shrunk to GTV only (CTV-3), and no margin is added to PTV-3 (Figure 1). Normal tissue structures, such as rectum, sigmoid colon, bladder, and small bowel in the pelvis are excluded from PTV as much as possible. If PTV-1 and PTV-2 overlapped normal tissues, priority is given to target coverage. However, in the first two clinical trials of C-ion RT (9403 and 9702) for squamous cell carcinoma of the uterine cervix, 18% of patients developed major gastrointestinal (GI) complications ([13](#_ENREF_13)). The dose to the GI tracts was limited to < 60 GyE according to DVH analysis, and this limitation had higher priority than the prescription to PTV-3 as final boost irradiation. Based on the results of previous trials, the doses to PTV-1, PTV-2 and PTV-3 were fixed at 39.0 GyE in 13 fractions, 15.0 GyE in 5 fractions and 18 GyE in 2 fractions, respectively, and the total dose to local tumor (GTV) was 72.0 GyE over 20 fractions. None of the patients received concurrent chemotherapy.

9-7 Study plan

This study designed the phase I/ II clinical trial. Phase I was designed to assess the safety for the first 6 patients. If some of the 6 patients develop grade 3 or higher non-hematological toxicities or grade 4 or higher hematological toxicities according to NCI-CTCAE version 3, reduction of the prescribed dose for the para-aortic region will be discussed by The Working Group of the Gynecological Tumor. After confirmation of safety, the study will move to phase II.

If 2 patients develop PALN failure without pelvic failure in this study, the continuation of this study will be discussed by The Working Group of the Gynecological Tumor.

9-8 Dose constraints

Rectum and sigmoid colon: 60GyE in 5 weeks

Small intestine: 50GyE in 5 weeks

Bladder: 1/3 of volume: 40GyE in 4 weeks; maximum: 70GyE in 5 weeks

Kidney: 1/2 of volume: 10GyE in 3 weeks; maximum: 30GyE in 3 weeks

9-9 Other therapy treatment during or after C-ion RT

Not applicable to this study, but treatment for adverse events of patients has no limitations.

10 Pathology

10-1 Tumor specimens before treatment

Working group pathologists reviewed the tumor specimens.

10-2 Tissue or tumor specimens after C-ion RT

When patients are suspected of recurrence after C-ion RT and undergo biopsy or salvage surgery, the tissues or tumor specimens after C-ion RT are confirmed by pathological examination at each hospital or NIRS.

11 Required evaluation

11-1 Pre-treatment evaluation

1. History and physical examination
2. Zubrod Performance Status
3. Cervical biopsy
4. Clinical examination
5. Blood cell counts
6. Chemistry profile
7. Urinalysis
8. Tumor markers (SCC, CEA etc.)
9. Diagnostic imaging: X-ray, CT, MRI, PET (if possible).
10. Cystoscopy and rectoscopy

11-2 Evaluation of observation and follow-up period

Ninety days after the start of C-ion RT is defined as the observation period. The period between the end of the observation period and 3 years after the registration of the final case is defined as the follow-up period.

11-2-1 Clinical examination during C-ion RT, observation period and follow-up period

Blood cell counts and chemistry profiles are performed every 2 weeks during C-ion RT, every 2 months during the observation period, and every 6 months during the follow-up period.

11-2-2 Evaluation during C-ion RT, observation period and follow-up period

Evaluation of normal tissue complications is conducted every 2 weeks during C-ion RT, monthly during the observation period, and every 6 months during the follow-up period according to “RTOG Acute Radiation Morbidity Scoring System”, “NCI Common Terminology Criteria for Adverse Events, Version 3.0” or “RTOG/EORTC Late Radiation Morbidity Scoring System”.

11-3 Evaluation of tumor response and failure

After the end of C-ion RT, CT or MRI for evaluation of initial tumor response will be performed during the observation period, and CT or MRI will be performed every 6 months during the follow-up period.

12 Expected adverse reaction, management of adverse reaction, and treatment modification

12-1 Expected adverse reaction

Acute and late adverse reactions, reported in“RTOG Acute Radiation Morbidity Scoring System”, “NCI Common Terminology Criteria for Adverse Events, Version 3.0” and “RTOG/EORTC Late Radiation Morbidity Scoring System”, are expected.

12-2 C-ion RT modification

If patients develop grade 3 or higher non-hematological toxicities or grade 4 or higher hematological toxicities during C-ion RT, The Working Group of the Gynecological Tumor will discuss and decide the reduction of the prescribed dose for C-ion RT and continuation of this trial.

12-3 Adverse reaction management

If patients develop acute and late adverse reactions that are reported in “RTOG Acute Radiation Morbidity Scoring System”, “NCI Common Terminology Criteria for Adverse Events, Version 3.0” and “RTOG/EORTC Late Radiation Morbidity Scoring System”, they will receive appropriate examination and treatment, and the attending physician must record them.

12-4 Unexpected adverse reaction

If patients develop unexpected adverse reactions of which the causal relationship of C-ion RT cannot be ruled out, The Working Group of the Gynecological Tumor will discuss this relationship and the attending physician will explain this to the patients.

13 Additional treatments after C-ion RT

Additional treatment for the tumor is not permitted before local or distant failure. After local or distant failure, there are no limits to additional treatment.

14 Clinical trial evaluation and report

14-1 The Working Group of the Gynecological Tumor

Clinical trial evaluation and report are checked by The Working Group of the Gynecological Tumor, which will meet on a semi-annual basis.

14-2 Clinical trial assessment

- Case registration
- Eligibility / ineligibility of each case
- Pathological findings of each case
- C-ion radiotherapy of each case
- Patient characteristics at registration of each case
- Status of acute and late toxicities in study
- Status of local or distant failure

15 Trial registration expiration

15-1 Expiration

When the number of registrations reaches the sample size and are checked by The Working Group of the Gynecological Tumor, registration for the trial will be concluded.

15-2 Termination of the trial

If an unexpectedly higher rate of grade 3 or higher acute or late toxicities develops in this study, the termination of this study will be discussed by The Working Group of the Gynecological Tumor. If The Working Group of the Gynecological Tumor decides on terminating the trial, the reasons for the termination are reported to the Network Committee for the Research Project with Heavy Ions at the National Institute of Radiological Sciences-Heavy Ion Medical Accelerator in Chiba. These are also explained to the patients.

16 Reporting requirements of adverse event / adverse reaction

16-1 Reporting requirements of adverse event / adverse reaction

If patients develop “serious adverse reaction” or “unexpected adverse event”, physicians have to report these to the Project Organizer and The Working Group of the Gynecological Tumor.

16-2 Reporting requirements by attending physicians

16-2-1 Rapid reporting

- Primary report: When attending physicians know of serious adverse reactions, they have to submit AE/AR rapid primary report on C-ion RT to the Project Organizer and The Working Group of the Gynecological Tumor within 72 hours.
- Secondary report: AE/AR report on C-ion RT and details of the case report must be submitted within 7 days.
- Tertiary report: completed AE/AR report on C-ion RT must be submitted within 15 days.
- Additional report: If additional report is needed after tertiary report, this needs to be submitted to the Project Organizer and The Working Group of the Gynecological Tumor.

16-2-2 Normal report

- Unexpected grade 3 adverse event, which is not listed in ‘12-1 Expected adverse reaction’, must be reported in a normal report.

17 Statistical considerations

17-1 Definition of endpoint and method of analysis

17-1-1 Local control

Local control is defined as no recurrence or no appearance of new lesion in PTV, and local control rate will be calculated by Kaplan-Meier method. Local recurrence is defined as evidence of tumor regrowth or recurrence in the treatment volume according to physical examination, CT, MRI, PET, and/or biopsy after treatment.

17-1-2 Survival duration

All failure times will be measured from the day of the start for C-ion RT to the date of failure. Recurrence and death from any cause will be counted for progression-free survival, and death from any cause will be counted for overall survival. These will be calculated by Kaplan-Meier method.

17-2 Handling cases for analysis

Rules for handling cases for analysis are according to “the General Rules for cancer research in Japanese by Japan Society of Clinical Oncology”.


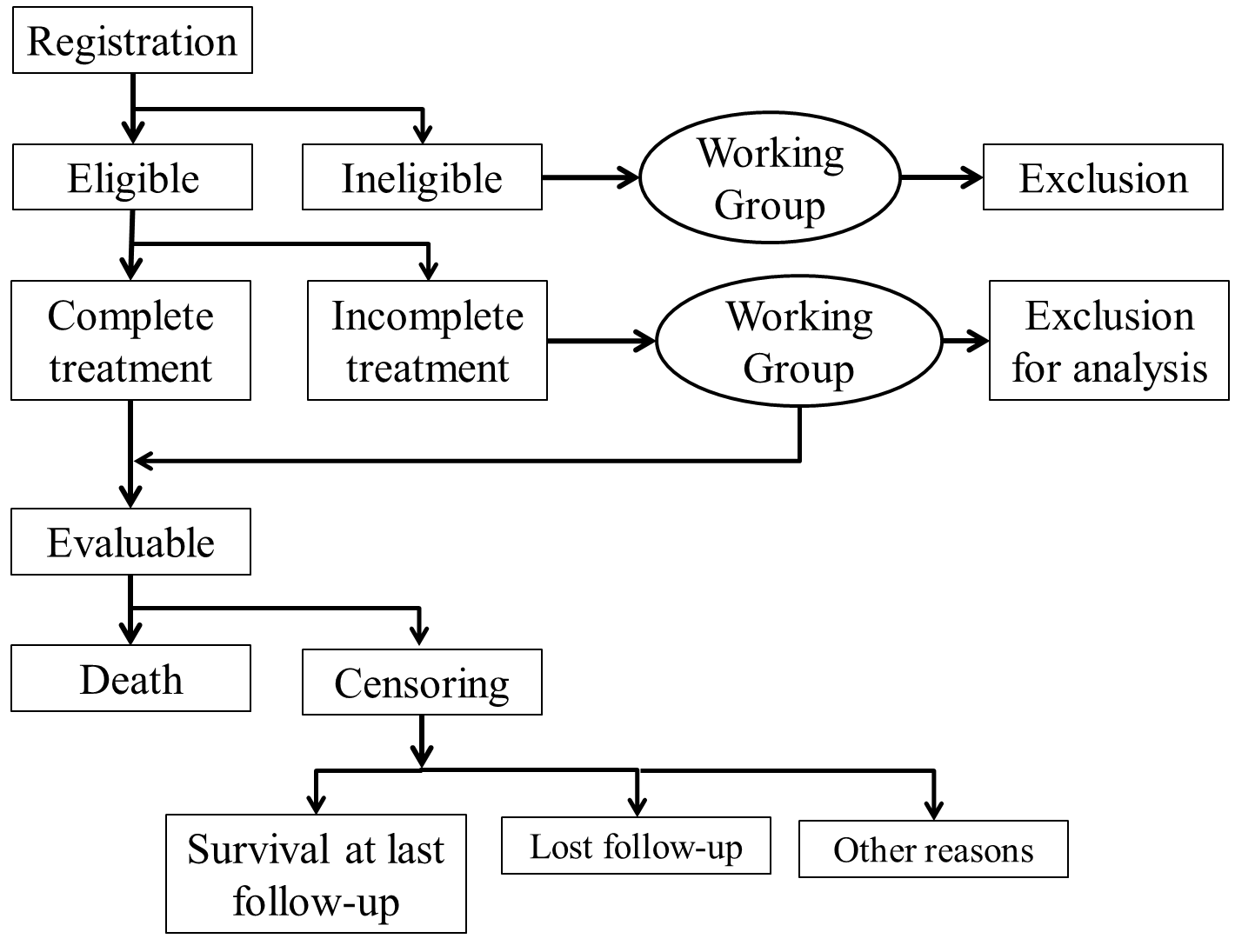


18 Study team

This study is supported by the Research Project with Heavy Ions at the National Institute of Radiological Sciences (NIRS). This treatment protocol was reviewed and approved by the NIRS Ethics Committee of Human Clinical Research and Network Committee for the Research Project with Heavy Ions at the National Institute of Radiological Sciences-Heavy Ion Medical Accelerator in Chiba.

18-1 Project leader

Michiya Suzuki

National Institute of Radiological Sciences; Special advisor

18-2 Project organizer

Shingo Kato

Research Center for Charged Particle Therapy, National Institute of Radiological Sciences

19 References

1. Eifel PJ, Winter K, Morris M, et al. Pelvic irradiation with concurrent chemotherapy versus pelvic and para-aortic irradiation for high-risk cervical cancer: An update of radiation therapy oncology group trial (rtog) 90-01. *J Clin Oncol* 2004;22:872-880.

2. Rose PG, Bundy BN, Watkins EB, et al. Concurrent cisplatin-based radiotherapy and chemotherapy for locally advanced cervical cancer. *N Engl J Med* 1999;340:1144-1153.

3. Whitney CW, Sause W, Bundy BN, et al. Randomized comparison of fluorouracil plus cisplatin versus hydroxyurea as an adjunct to radiation therapy in stage iib-iva carcinoma of the cervix with negative para-aortic lymph nodes: A gynecologic oncology group and southwest oncology group study. *J Clin Oncol* 1999;17:1339-1348.

4. Green JA, Kirwan JM, Tierney JF, et al. Survival and recurrence after concomitant chemotherapy and radiotherapy for cancer of the uterine cervix: A systematic review and meta-analysis. *Lancet* 2001;358:781-786.

5. Nakano T, Kato S, Ohno T, et al. Long-term results of high-dose rate intracavitary brachytherapy for squamous cell carcinoma of the uterine cervix. *Cancer* 2005;103:92-101.

6. Lagasse LD, Creasman WT, Shingleton HM, et al. Results and complications of operative staging in cervical cancer: Experience of the gynecologic oncology group. *Gynecol Oncol* 1980;9:90-98.

7. Varia MA, Bundy BN, Deppe G, et al. Cervical carcinoma metastatic to para-aortic nodes: Extended field radiation therapy with concomitant 5-fluorouracil and cisplatin chemotherapy: A gynecologic oncology group study. *Int J Radiat Oncol Biol Phys* 1998;42:1015-1023.

8. Niibe Y, Nakano T, Ohno T, et al. Prognostic significance of c-erbb-2/her2 expression in advanced uterine cervical carcinoma with para-aortic lymph node metastasis treated with radiation therapy. *Int J Gynecol Cancer* 2003;13:849-855.

9. Pearcey R, Brundage M, Drouin P, et al. Phase iii trial comparing radical radiotherapy with and without cisplatin chemotherapy in patients with advanced squamous cell cancer of the cervix. *J Clin Oncol* 2002;20:966-972.

10. Rotman M, Pajak TF, Choi K, et al. Prophylactic extended-field irradiation of para-aortic lymph nodes in stages iib and bulky ib and iia cervical carcinomas. Ten-year treatment results of rtog 79-20. *JAMA* 1995;274:387-393.

11. Haie C, Pejovic MH, Gerbaulet A, et al. Is prophylactic para-aortic irradiation worthwhile in the treatment of advanced cervical carcinoma? Results of a controlled clinical trial of the eortc radiotherapy group. *Radiother Oncol* 1988;11:101-112.

12. Portelance L, Chao KS, Grigsby PW, et al. Intensity-modulated radiation therapy (imrt) reduces small bowel, rectum, and bladder doses in patients with cervical cancer receiving pelvic and para-aortic irradiation. *Int J Radiat Oncol Biol Phys* 2001;51:261-266.
